# Integrin β3-mediated platelet extracellular vesicle adhesion facilitates vascular smooth muscle cell dysfunction in postinjury intimal hyperplasia

**DOI:** 10.7150/ijbs.101391

**Published:** 2025-03-03

**Authors:** Fei Zhuang, Zhi-tong Liu, Guo Zhou, Feng Liang, Ying-hua Wang, Long Chen, Wei-feng Zhang, Ling-hong Shen, Yan-qiao Lu, Huan-huan Huo, Xin Shi, Liang Fang, Ben He

**Affiliations:** 1 Department of Cardiology, Shanghai Chest Hospital, School of Medicine, Shanghai Jiao Tong University, Shanghai 200030, China.; 2 Department of Cardiac Surgery, Shanghai Chest Hospital, Shanghai Jiao Tong University School of Medicine, Shanghai 200030, China.

**Keywords:** platelet-derived extracellular vesicles, intimal hyperplasia, vascular smooth muscle cell, SPP1, ITGβ3

## Abstract

Vascular smooth muscle cell (VSMC) dysfunction is a critical pathological process in postinjury intima hyperplasia. This process is driven by the adherence and accumulation of platelet-derived extracellular vesicles (PEVs) released from activated platelets to VSMCs at the site of injured intima. However, the precise mechanism remains unclear. Thus, the present study aimed to investigate how PEVs adhere to VSMCs and facilitate VSMC dysfunction in postinjury intimal hyperplasia. Morphological results confirmed that PEVs led to VSMC dysfunction and intimal hyperplasia. Integrated single-cell and proteomic analysis revealed that increased secreted phosphoprotein 1 (SPP1) expression in VSMCs played a central role in this process, possibly by mediating PEV adhesion to VSMCs and activating the focal adhesion kinase (FAK)/phosphoinositide 3 kinase (PI3K)/protein kinase B (Akt) axis. In addition, integrin beta 3 (ITGβ3, CD61) on PEVs, with increased expression under pathological conditions, was predicted to interact with SPP1. Co-immunoprecipitation (Co-IP) analysis further confirmed that ITGβ3 interacted with SPP1, thereby activating the FAK/PI3K/AKT phosphorylation and promoting PEV adhesion. Of note, blocking ITGβ3 expression on PEVs reduced PEV adhesion and intimal hyperplasia. Thus, ITGβ3-SPP1-mediated PEV adhesion to VSMCs may be a novel mechanism in intimal hyperplasia, which proposed to be critical for vascular homeostasis.

## 1. Introduction

Vascular endothelial injury usually occurs after certain vascular surgeries, including coronary artery bypass grafting, carotid endarterectomy, and stent placement for arterial occlusive disease. Although such treatments improve vascular patency, intimal hyperplasia and restenosis after vascular surgery have become the primary limiting factors impacting their clinical efficacy. In this context, vascular smooth muscle cell (VSMC) dysfunction, along with abnormal phenotypic switching, proliferation, and migration 1, 2, is a crucial factor in the development of intimal hyperplasia. Therefore, elucidating the underlying mechanisms driving VSMC dysfunction in intimal hyperplasia is crucial for enhancing the cure rate of cardiovascular diseases.

Numerous studies have indicated many factors, such as lipoproteins 3, noncoding RNAs 4, 5, and reactive oxygen species 6, 7 regulate VSMC dysfunction. Platelets have also been confirmed to participate in VSMC dysfunction 8, 9. Once endothelial cells (ECs) are damaged, the exposure of underlying collagen (mainly collagen I) triggers a rapid accumulation and activation of platelets, ultimately leading to VSMC dysfunction and most likely resulting in neointimal hyperplasia or restenosis 10. Although studies have confirmed that platelets regulate VSMC dysfunction and participate in intimal hyperplasia, the precise underlying mechanism is unclear.

Platelet extracellular vesicles (PEVs), derived from activated platelets, carry various types of biological information, including proteins and microRNAs, which vary depending on the response of platelets to different agonists 11-13. PEV-mediated intercellular communication has been recognized as a key factor in the advancement of vascular pathology 14, 15. In hypertension, activated PEVs enhance the abnormal proliferation of ECs by transporting miR-142-3p from activated platelets to ECs 16. The interaction between neutrophils and ECs is also promoted by activated PEVs, which facilitates this process through the direct transfer of the chemokine (C-C motif) ligand 5 to ECs 17. When the vascular intima is injured, activated PEVs increase Col8a1 secretion and vascular stiffness by transferring miR-92a-3p to VSMCs, thereby facilitating postinjury intimal hyperplasia 18. In addition, PEVs upregulate calcium oscillations via TRPV4 and increase abnormal VSMC migration, simultaneously aggravating neointimal hyperplasia 19. Although existing research has revealed that PEVs participate in vascular pathology, the underlying mechanisms of PEVs in VSMC dysfunction and neointimal hyperplasia still remain poorly understood.

During vascular intimal injury, the adherence and accumulation of PEVs to VSMCs is a crucial pathological event. EVs regulate target cell function, either by interacting with proteins on the cell surface or being internalized by recipient cells. This is mediated by various mechanisms ranging from membrane fusion to uptake 20, 21. These processes are currently the least understood mechanisms in EV-mediated intercellular communication. Because PEVs have been shown to promote VSMC dysfunction and neointimal hyperplasia, exploring possible mechanisms regulating PEV adhesion to VSMCs will be beneficial for ameliorating intimal hyperplasia.

The secreted phosphoprotein 1 (SPP1), known as osteopontin, has been shown to drive vascular calcification within advanced atherosclerosis plaques by fostering VSMC transformation to osteoblast-like cells 22, and aggravate fibrosis in atherosclerotic coronary arteries by enhancing fibroadipogenic progenitor cell migration and proliferation 23. Meanwhile, SPP1 inhibition in pulmonary artery smooth muscle cells using mesenchymal stromal cell-derived exosomes has been shown to attenuate pulmonary vascular remodeling 24. By contrast, elevated SPP1 expression in macrophages and aortic tissue caused vascular inflammation and remodeling by triggering the adhesion and migration of platelets into the abdominal aorta, contributing to the formation of intraluminal thrombus 25. Although SPP1 regulates vascular homeostasis and platelet adhesion, whether SPP1 regulates PEV adhesion to VSMCs during intimal hyperplasia progression remains unclear.

In the present study, using a rat model of carotid artery injury *in vivo* and PEV-induced VSMC dysfunction *in vitro*, the underlying mechanism of PEVs in VSMC dysfunction and intimal hyperplasia were investigated. Single-cell sequencing combined with bulk proteomics indicated SPP1 played a central role in PEV-induced VSMC dysfunction and intimal hyperplasia, possibly by mediating PEV adhesion to VSMCs. The present study aimed to explore the potential targets on PEVs involved in this process and offer novel potential therapeutic targets to prevent postinjury intimal hyperplasia.

## Materials and Methods

### Rat model of carotid artery intimal injury

All animal care and experiments in this study were performed according to the Animal Management Rules of China (55, 2001, Ministry of Health, China). The study was approved by the Institutional Animal Care and Use Committee at Shanghai Chest Hospital, which is affiliated to Shanghai Jiaotong University School of Medicine.

Male Sprague Dawley (SD) rats aged 8-10 weeks were used for establishing the intimal vascular injury model 26. In brief, the SD rats were anesthetized via isoflurane inhalation and the left carotid artery was bluntly separated. Subsequent to the ligation of the internal and external carotid arteries, and occlusion of the occipital and proximal common carotid arteries, an inflatable balloon device (Edwards Lifesciences, 12A0602F) was introduced into the common carotid artery via transverse arteriotomy in the external carotid artery while the undamaged contralateral right carotid artery was considered as the control. The arteries were collected after a period of either 2 or 4 weeks for subsequent experiments.

### Single-cell RNA sequencing

The carotid arteries of 10 male SD rats were obtained and digested into single cell. Subsequently, the 10× Genomics Platform was utilized to perform RNA sequencing from individual cells. In brief, microfluidic methods were employed to encapsulate individual cells along with beads containing cell barcodes within droplets. mRNA within the lysed cells was captured by the cell barcodes attached to the beads, resulting in the creation of single-cell gel beads-in-emulsion (GEMs). Additionally, the mRNA in droplets underwent reverse transcription to facilitate the creation of a cDNA library. The identification of source samples for the target sequence was subsequently achieved via library sequencing using a distinct sample index.

### Hematoxylin/eosin and immunofluorescence staining

Following collection, the arteries were preserved in 4% paraformaldehyde, subjected to dehydration with 30% sucrose solution, embedded, and sectioned into 8-10-μm slices. Subsequently, HE staining was performed, as previously described 27.

For immunofluorescence staining, the paraffin-embedded sections were subjected to permeabilization with 0.3% Triton X-100 for 10-15 min. Next, 10% goat serum was used to blot the sections for 30 min to reduce nonspecific binding, and the samples were subsequently incubated with primary antibodies at 4°C overnight. The primary antibodies were against SPP1 (1:100, Proteintech), ITGβ3 (1:100, Proteintech), and α-smooth muscle actin (α-SMA; 1:100, Cell Signaling Technology). After washing with phosphate-buffered saline (PBS), the sections were incubated with secondary antibodies at room temperature for 1 h. The secondary antibodies were Alexa Fluor 568-conjugated IgG (1:1000, Invitrogen) and Alexa Fluor 488-conjugated IgG (1:1000, Invitrogen). Finally, 4, 6-diamidino-2-phenylindole (DAPI) was used for the staining of cell nucleus for 5 min, and confocal laser scanning microscopy (Fluoview 1000, Olympus) was performed to capture fluorescence images.

### PEV isolation and identification

PEVs were collected from activated platelets *in vitro*, as previously described 16, 19. In brief, whole blood samples were obtained from the abdominal aorta of anesthetized 8-10-week-old male SD rats via a 10-mL syringe that contained 100 μL/mL anticoagulant (0.1 g/mL PGE1, 1 U apyrase, and 2.94% sodium citrate). Platelet-rich plasma was collected by centrifugation at 1500 rpm for 10 min, and platelets were subsequently separated at 2800 rpm for 15 min. Tyrode solution was used to resuspend and wash the extracted platelets. In addition, human peripheral blood samples were obtained from in-sent restenosis patients or healthy volunteers without cardiovascular disease; platelets from these samples were isolated in the same manner as above. Following this, the platelets were activated with 1 U/mL collagen I for 60 min at 37°C and subsequently separated through centrifugation at 5000 rpm for 15 min. Finally, the remaining supernatant was subjected to centrifugation at 20500 ×*g* for 90 min to isolate PEVs, which were resuspended in a medium purified using a 0.1-μm filter.

The isolated PEVs were diluted in PBS, and NanoSight analyzer was employed to assess the quantity and size distribution of PEVs. The origin of the generated particles was evaluated using the EV markers CD9, CD63, and TSG101 and the platelet-derived EV marker CD41 before subsequent use.

### VSMC culture and PEV treatment

Primary VSMCs isolated from the carotid artery of 6-8-week-old male SD rats were cultured in Dulbecco's modified Eagle medium (DMEM, Gibco) containing 10% fetal bovine serum (FBS, Gibco) and characterized using antibodies against α-SMA (1:100, Cell Signaling Technology), a VSMC marker. Cells from passages 4-7 at >95% confluency were used for subsequent experiments.

To block ITGβ3, PEVs were incubated with a monoclonal antibody against ITGβ3 (554951, BD Pharmingen™). For PEV treatment, VSMCs were seeded at 50% density and treated with PEVs (10^9^/mL) for either 1 or 24 h after being serum-starved overnight. Following surgery, the 8-10-week-old male SD rats were administered tail vein injections of either normal saline (control) or PEVs (6 × 10^9^/mL) on alternating days, and the carotid artery tissue was collected on day 14, as previously described 19.

### Proliferation assays

Both Edu and CCK-8 assays were used for detecting VSMC proliferation. For Edu immunostaining or flow cytometry *in vitro*, VSMCs were seeded at 50% density and treated with PEVs (10^9^/mL) for 24 h after being serum-starved overnight. Based on the manufacturer's protocol, PEV-induced VSMC proliferation was evaluated using the BeyoClick™ Edu-488 kit (Beyotime). A fluorescence microscope (Leica710) and flow cytometer (BD FACS CantoII), respectively, were used to assess the results.

For the CCK-8 assay, VSMCs were treated with PEVs (10^9^/mL) for 24 h after being serum-starved overnight. According to the test kit instructions, CCK-8 solution (Beyotime) was added into the supernatant and incubated with VSMCs for 1 h. OD values were recorded using an ELISA plate reader (Bio-Rad 680) at 450 nm, with the data normalized to the control group.

### Scratch wound healing assay

To evaluate the effect of PEVs on the migration of VSMCs *in vitro*, VSMCs were seeded at 50% density and treated with PEVs (10^9^/mL) for 24 h. Then, a sterile 10-μL pipette tip was utilized to make a scratch line across the VSMC monolayer. The cells were imaged before and after PEV incubation using a microscope (Olympus, IX-71), and the wound area was analyzed using the ImageJ software (NIH, USA). Cell migration was analyzed using the formula (S0 - St) / S0 (S0 represents the wound area at the initial time point and St represents the wound area at 24 h).

### PEV adhesion to VSMCs

To assess the binding of PEVs to VSMCs, PEVs were treated with the PKH-26 red fluorescent dye (Sigma-Aldrich) following the manufacturer's instructions and then resuspended in DMEM purified using a 0.1-μm filter. Subsequently, the PKH-26-labeled PEVs were incubated with VSMCs for 1 h. Following two rinses with PBS, the cells were subjected to fixation with 4% PFA for 15 min, after which DAPI was used to stain the nucleus for 5-10 min. The adhesion of PEVs to VSMCs was examined under a fluorescence microscope (Zeiss LSM 710), and the average intensity of the fluorescent signal was quantified utilizing the ImageJ software.

### siRNA transfection

For RNA interference experiments, VSMCs were initially plated in 6-well culture dishes at a density of 50%. After attachment, VSMCs were subjected to transfection with siRNAs specific to SPP1 or negative control (NC) for 48 h based on the protocol provided by GenePharma. The forward and reverse sequences for the SPP1-targeting siRNAs were 5′-GGA UGA ACC AAG CGU GGA ATT-3′ and 5′-UUC CAC GCU UGG UUC AUC CTT-3′. The NC siRNA sequences were 5′-UUC UCC GAA CGU GUC ACG UTT-3′ and 5′-ACG UGA CAC GUU CGG AGA ATT-3′.

### Western blotting

Proteins (15-20 μg) in the samples were separated via 10% sodium dodecyl sulfate-polyacrylamide gel electrophoresis and then transferred onto polyvinylidene fluoride (PVDF) membranes (Hybond, Amersham). Following the blocking of nonspecific protein binding with 5% nonfat milk at room temperature for 1 h, the appropriate primary antibodies against SPP1 (1:2,000, Proteintech), phospho-PI3K p85 (Tyr458)/p55 (Tyr199) (1:1000, Cell Signaling Technology), phospho-Akt (Ser473) (1:1,000, Cell Signaling Technology), phospho-FAK (Y397) (1:1,000, Cell Signaling Technology), ITGβ3 (1:2,000, Proteintech), CD9 (1:2,000, Proteintech), CD63 (1:2,000, Proteintech), TSG101 (1:2,000, Proteintech), CD41 (1:5,000, Proteintech), and GAPDH (1:5,000, Cell Signaling Technology) were added to the PVDF membranes and incubated overnight at 4°C. Next, the samples were incubated in buffers containing secondary antibodies conjugated with horseradish peroxidase (HRP) for 2 h at room temperature. The target proteins were detected using a chemiluminescence method facilitated by an enhanced chemiluminescence kit (Thermo Fisher Scientific).

### Gene overexpression

Adenovirus containing the pAdEasy-EF1-ITGβ3-CMV-mCherry, pAdEasy-EF1-NC-CMV-mCherry, pAdEasy-EF1-SPP1-CMV-EGFP, and pAdEasy-EF1-NC-CMV-EGFP plasmids were constructed. Primary VSMCs were seeded at 30% confluence and infected with the adenovirus in growth media containing 5 µg/mL polybrene. Growth media were replaced before the infection, and infected cells were harvested at 48 h for subsequent analysis. SPP1 along with full-length ITGβ3 and its mutant vectors were also constructed and cotransfected into 293T cells for subsequent experiments.

### Co-immunoprecipitation assay

Proteins from transfected cells were extracted with a cold lysis solution for 30 minutes at 4°C. Next, equal quantities of the extracted proteins were combined with the respective SPP1, ITGβ3, or IgG beads and incubated at 4°C overnight. After washing the beads three times with the lysis solution, they were subjected to elution with 2× SDS loading buffer and heated at 95°C for 10 min. Finally, the supernatant was subjected to western blotting analysis.

### Bioinformatics

To perform the Gene Ontology (GO) enrichment analysis of the differentially expressed genes, a comprehensive multi-omics data analysis tool, OmicsBean, was utilized. In addition, the pathway classification of the candidate genes was performed using the Kyoto Encyclopedia of Genes and Genomes (KEGG). The String tool's “connect” feature was used to forecast differentially expressed genes in PEVs that possibly interact with SPP1. In addition, differentially expressed genes in PEVs possibly involved in PI3K activation were predicted using the Ingenuity Pathway Analysis (IPA) tool (version: 65367011).

### Statistical analysis

Each experiment in this study was performed at least thrice, with all data represented as the mean ± standard error. Comparisons between two groups were assessed using Student's t-test. The Kolmogorov-Smirnov test was used to evaluate the Gaussian distribution of the data. For paired data that followed a Gaussian distribution, the paired t-test was utilized. Meanwhile, the unpaired t-test was applied to analyze the unpaired data conforming to a Gaussian distribution. For the paired data that lacked a Gaussian distribution or data with a sample size of <5, a Wilcoxon matchedpairs signed rank test was used. All statistical analyses were performed using MS Excel and GraphPad Prism 8, and P-value <0.05 was considered statistically significant.

## Results

### PEVs induced VSMC dysfunction and facilitated postinjury intimal hyperplasia

To evaluate the progression of vascular remodeling after intimal injury, immunofluorescence staining was used to measure the extent of intimal hyperplasia and assess changes in vascular morphology in the carotid artery at 2 weeks. The results revealed that the intima was thickened and VSMCs, labeled with α-SMA, were increased in the injured carotid artery compared with the contralateral common carotid artery (Figure S1A), indicating intimal hyperplasia was obviously formed after intimal injury and VSMC dysfunction played vital roles in this process.

**Figure 1:**
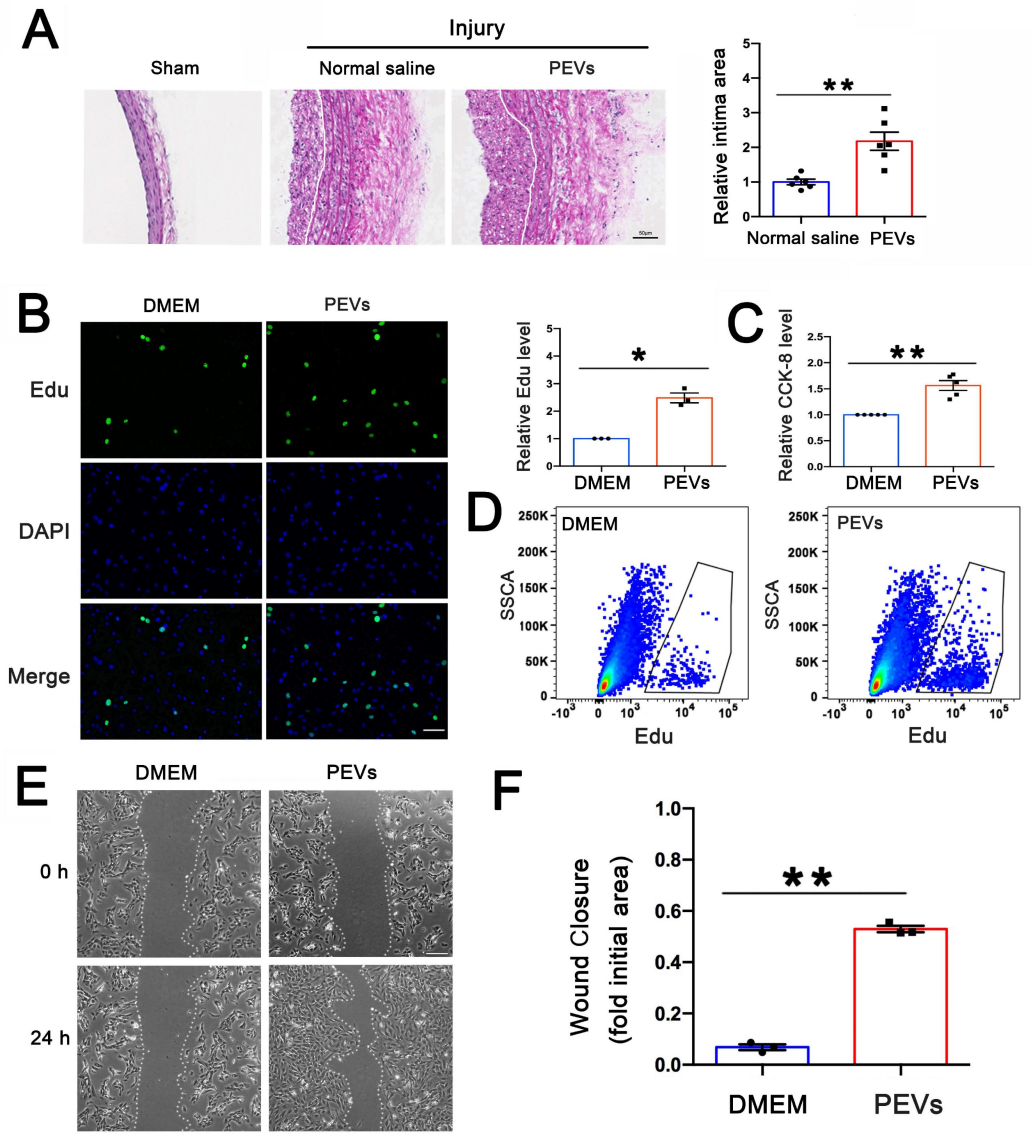
** PEVs induced VSMC dysfunction and facilitated postinjury intimal hyperplasia.** (A) PEVs and normal saline (control) were respectively injected into the rat model after intimal injury. White line represents the inner elastic fibers. HE staining revealed that PEV administration promoted intimal hyperplasia in the injured carotid arteries. Scale bar = 50 μm. (B-C) VSMCs were treated with PEVs or DMEM (control) for 24 h after being serum-starved overnight. Edu was used to evaluate the effect of PEVs on VSMC proliferation, and the results were obtained using immunofluorescence staining (green) and flow cytometry, respectively. The results showed that PEVs promoted VSMC proliferation. Cell nucleus was stained with DAPI (blue). Scale bar = 50 μm. (D) CCK-8 was used to analyze the role of PEVs in VSMC proliferation, and the results confirmed that PEVs increased VSMC proliferation compared with the control (DMEM). (E-F) DMEM and PEVs were incubated with VSMCs for 24 h respectively, and the migration of PEV-induced VSMCs was assessed using the wound healing assay. The data revealed that PEVs increased VSMC migration. Scale bar = 200 μm.

After vascular intimal injury, the initial attachment and accumulation of platelets to the exposed VSMCs represented a critical pathological process. The platelets exposed to collagen underwent activation and subsequently released various heterogeneous PEVs. Nanoparticle tracking analysis (NTA), used to determine the sizes of these PEVs, revealed four distinct size peaks at 83, 113, 211, and 305 nm (Figure S1B). Electron microscopy confirmed the EV morphology and size (Figure S1C). In addition, western blotting revealed that EV markers (CD9, CD63, and TSG-101) and the platelet-specific marker CD41 were present on the extracted sample (Figure S1D), and the expression of calnexin, expressed in the endoplasmic reticulum of cells not in EVs, was used as a negative control (Figure S1E). All these results suggested the purity of the extraced PEVs, which could be used in the following experiments. To evaluate the role of PEVs in intimal hyperplasia, PEVs (6×10^9^ per ml) derived from male SD rats or normal saline were injected into the 8-10-week-old male SD rat tail vein on alternate days for 2 weeks after intimal injury, and the injured carotid artery was obtained on day 14. As shown in Figure 1B, HE staining revealed that PEV administration promoted postinjury intimal hyperplasia.

The abnormal migration and proliferation of VSMCs played vital roles in postinjury intimal hyperplasia. To evaluate the effect of PEVs on VSMC dysfunction, PEVs were incubated with primary VSMCs isolated from the carotid artery of 6-8-weeks-old SD rats *in vitro*. The influence of PEVs on the proliferation of VSMCs was assessed using both Edu and CCK-8 assays, and the results revealed that PEVs enhanced VSMC proliferation (Figures 1C-E). In addition, the scratch wound healing assay revealed that exposure to PEVs increased VSMC migration (Figures 1F-G). These results suggest that PEVs induce VSMC dysfunction and facilitate postinjury intimal hyperplasia after intimal injury in the carotid artery.

### SPP1^high^ VSMC subpopulation in intima hyperplasia may be involved in focal adhesion and PI3K-AKT pathway

**Figure 2:**
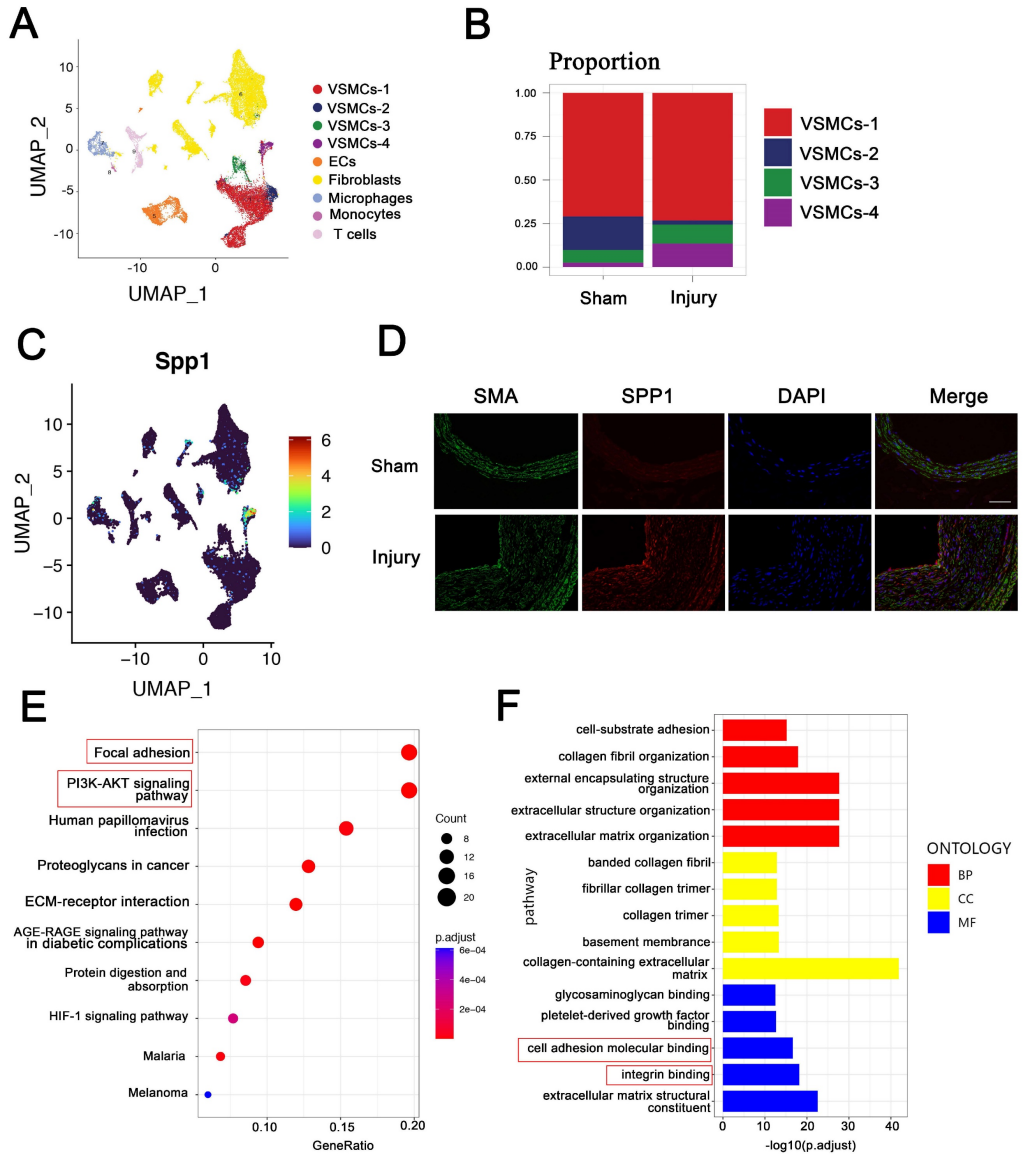
** SPP1^high^ VSMC subpopulation in intima hyperplasia may be involved in focal adhesion and PI3K-AKT pathway.** ScRNA-seq was used to analyze the profile of injured and self-contralateral common carotid artery (control). (A) UMAP classified VSMCs into four distinct clusters, including VSMC-1, VSMC-2, VSMC-3, and VSMC-4. (B) Proportion of VSMCs-4 was significantly increased after injury. (C) SPP1 was highly expressed in VSMCs-4, and VSMC-4 was referred to as SPP1^high^ VSMC subpopulation. (D) Immunofluorescence analysis revealed increased SPP1 expression in VSMC in intimal hyperplasia. (E) KEGG analysis indicated that focal adhesion and PI3K-AKT signaling pathway play crucial roles in SPP1^high^ VSMC subpopulation. (F) Molecular function analysis suggested that SPP1^high^ VSMCs subpopulation participates in cell adhesion molecular binding and integrin binding.

To characterize VSMC changes in intimal hyperplasia, scRNA-seq was used to analyze the profiles from the injuried and self-contralateral common carotid arteries. Uniform manifold approximation and projection (UMAP) classified VSMCs into four distinct clusters: VSMC-1, VSMC-2, VSMC-3, and VSMC-4 (Figure 2A). The proportion of VSMC-4 subpopulation was markedly increased in intimal hyperplasia (Figure 2B). SPP1 was highly expressed in the cells of the VSMC-4 cluster, which have been subsequently referred to as SPP1^high^ VSMC subpopulation (Figure 2C). Immunofluorescence staining confirmed the upregulated expression of SPP1 in intimal hyperplasia (Figure 2D). These results suggest that SPP1^high^ VSMC subpopulation participate in this pathological process.

To explore the role of SPP1^high^ VSMC subpopulation, KEGG analysis was performed, and the results suggested that SPP1^high^ VSMCs are involved in focal adhesion and PI3K-AKT signaling pathway (Figure 2E). More importantly, these pathways are known as essential mediators in intimal hyperplasia 28, 29. Molecular function analysis further indicated that SPP1^high^ VSMC subpopulation may be involved in cell adhesion molecular binding and integrin binding (Figure 2F). Importantly, SPP1 interactes with integrin involved in cell adhesion 30, and PEV adhesion to target cells is an important step in EVs-based intercellular communication. Overall, the results suggested SPP1, as a candidate molecule in VSMCs, may regulate PEV adhesion and PI3K-AKT pathway in intimal hyperplasia.

### SPP1 expression and FAK/PI3K/AKT phosphorylation were upregulated in PEV-treated VSMCs

**Figure 3:**
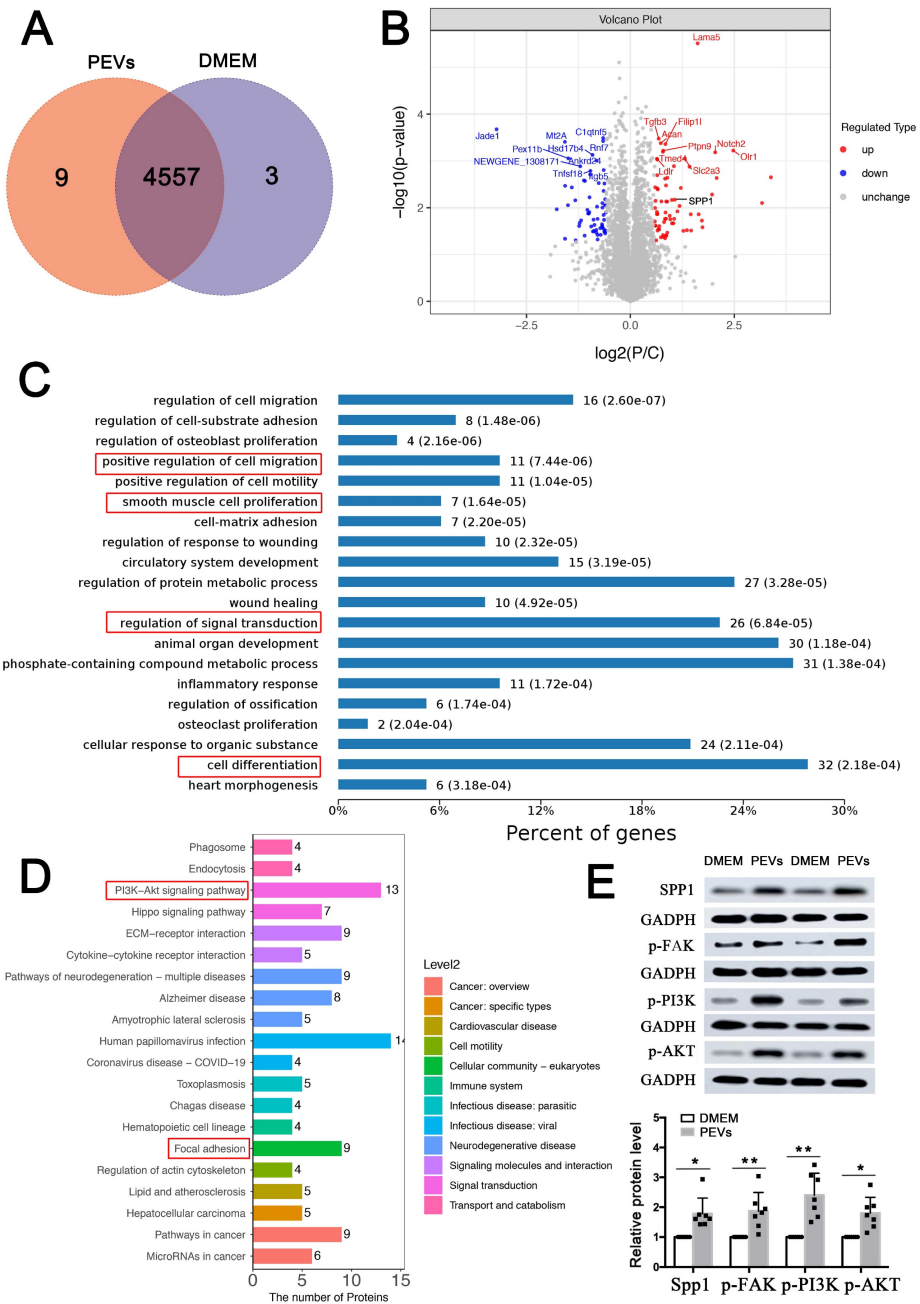
** SPP1 expression and FAK/PI3K/AKT phosphorylation were upregulated in PEV-treated VSMCs.** (A) Comparative proteomic analysis was applied to analyze PEV or DMEM (control) treated VSMCs. Venn diagram showing 4,557 proteins commonly expressed in both the PEV and control groups, with 9 expressed only in the PEV group and 3 expressed only in the DMEM group. (B) Volcano plot compared the levels of proteins in the control and PEV groups; 69 proteins, including SPP1, were significantly upregulated in the PEV group and 60 proteins were downregulated in the PEV group (P < 0.05 and fold change > 2). (C) Biological process analysis revealed that 129 differentially expressed proteins were associated with cell proliferation, migration, and differentiation. (D) KEGG analysis suggested that the differentially expressed proteins participate in focal adhesion and PI3K-AKT pathway. (E) Western blotting revealed increased SPP1 expression and p-FAK, p-PI3K, and p-AKT activation in PEV-treated VSMCs compared with the DMEM group.

In order to further verify the mechanism of PEVs regulating VSMC dysfunction *in vitro*, proteomic analysis was then performed. As shown in the Venn diagram, 9 proteins were exclusively expressed in PEV-treated VSMCs and 3 proteins were expressed only in the DMEM (control) group. Moreover, 4557 proteins were identified in these two groups (Figure 3A). Of these, 69 proteins, including SPP1, were upregulated, and 60 were downregulated in PEV-treated VSMCs compared with the control group (P < 0.05 and fold change > 2; Figure 3B, Supplementary Table S1).

Biological process and KEGG analyses used to analyze these 129 differentially expressed proteins revealed that the proteins positively regulated cell proliferation, migration, and differentiation (Figure 3C), consistent with the results of PEV-induced VSMC dysfunction in Figures 1C-E. In addition, PEV-induced differentially expressed proteins were predicted to participate in signal transduction. KEGG pathway analysis suggested that the proteins participate in focal adhesion and PI3K-AKT pathway (Figure 3D), which confirmed the single-cell analysis results of SPP1^high^ VSMCs. Given that, the role of SPP1 in focal adhesion and PI3K-AKT pathway was then explored. Since FAK was the upstream of PI3K-AKT pathway and played necessary role in focal adhesion pathway, the activation of p-FAK, p-PI3K, and p-AKT in PEV-treated VSMCs was analyzed. Western blotting revealed significantly increased SPP1 expression as well as p-FAK, p-PI3K, and p-AKT activation in PEV-treated VSMCs compared with the control group (Figure 3E). Meanwhile, there were no obvious difference in the total FAK (T-FAK), toal PI3K (T-PI3K), and total AKT (T-AKT) expression level between these two groups (Figure S2). Thus, further experiments focused on the role of SPP1, especially in mediating PEV adhesion and VSMC dysfunction.

### SPP1 knockdown inhibited PEV adhesion and PEV-induced VSMC dysfunction via FAK/PI3K/AKT phosphorylation

**Figure 4:**
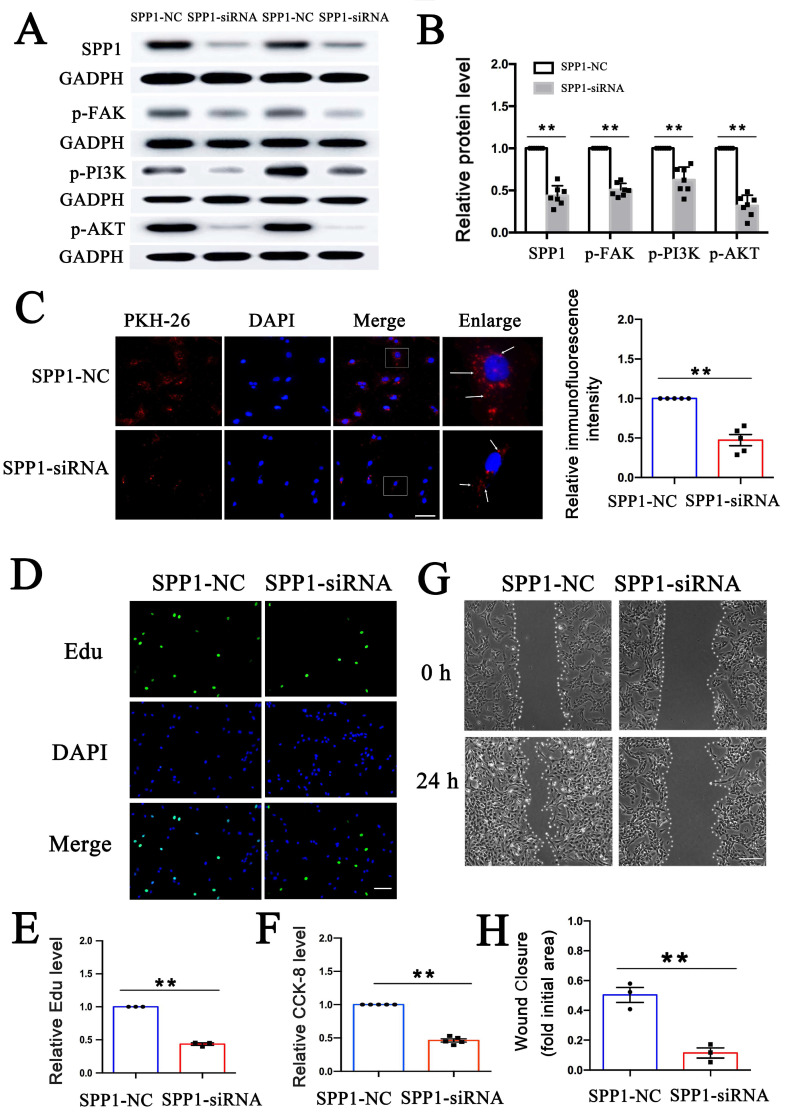
** SPP1 knockdown inhibited PEV adhesion and PEV-induced VSMC dysfunction via FAK/PI3K/AKT phosphorylation.** (A-B) SPP1-siRNA, transfected into VSMCs before PEV treatment, significantly inhibited SPP1 expression and FAK/PI3K/AKT phosphorylation in VSMCs. (C) Compared with SPP1-NC, SPP1-siRNA transfection in VSMCs significantly decreased PEV adhesion to VSMCs. PEVs were stained with PKH-26 (red) and VSMC nucleus was labeled with DAPI (blue). Scale bar = 20 μm. (D-E) Edu was used to evaluate the effect of SPP1-siRNA on PEV-induced VSMC proliferation, and the results were detected via immunofluorescence staining (green). The results showed that SPP1-siRNA suppressed PEV-induced VSMC proliferation. VSMC nucleus was stained with DAPI (blue). Scale bar = 20 μm. (F) CCK-8 results revealed that SPP1-siRNA decreased PEV-induced VSMC proliferation. (G-H) Scratch wound healing analysis revealed that SPP1-siRNA suppressed VSMC migration. Scale bar = 200 μm.

To explore whether PEV adhesion and the effect of PEVs on FAK/PI3K/Akt phosphorylation/activation are dependent on SPP1, SPP1-targeting siRNA was transfected into VSMCs prior to PEV treatment to knock down SPP1 expression. FAK/PI3K/Akt phosphorylation and PKH-26-labeled PEV adhesion to VSMCs were then assessed. The results revealed that SPP1-siRNA inhibited FAK/PI3K/Akt phosphorylation (Figures 4A-B) as well as PEV adhesion to VSMCs (Figure 4C). Besides, the expression level of T-FAK, T-PI3K, and T-AKT in SPP1-siRNA group showed no significant difference with that in the SPP1-NC group (Figure S3).

The role of SPP1 in PEV-induced VSMC proliferation was then assessed using EdU and CCK-8 assays. Both the EdU immunofluorescence and CCK-8 assay results showed that SPP1-siRNA significantly suppressed VSMC proliferation (Figures 4D-F). SSP1-siRNA also significantly suppressed PEV-induced VSMC migration (Figures 4G-H). These findings suggest that SPP1 in VSMCs is essential for FAK/PI3K/AKT phosphorylation, PEV adhesion, and VSMC dysfunction triggered by PEVs.

### ITGβ3 on PEVs was the upstream of SPP1

**Figure 5:**
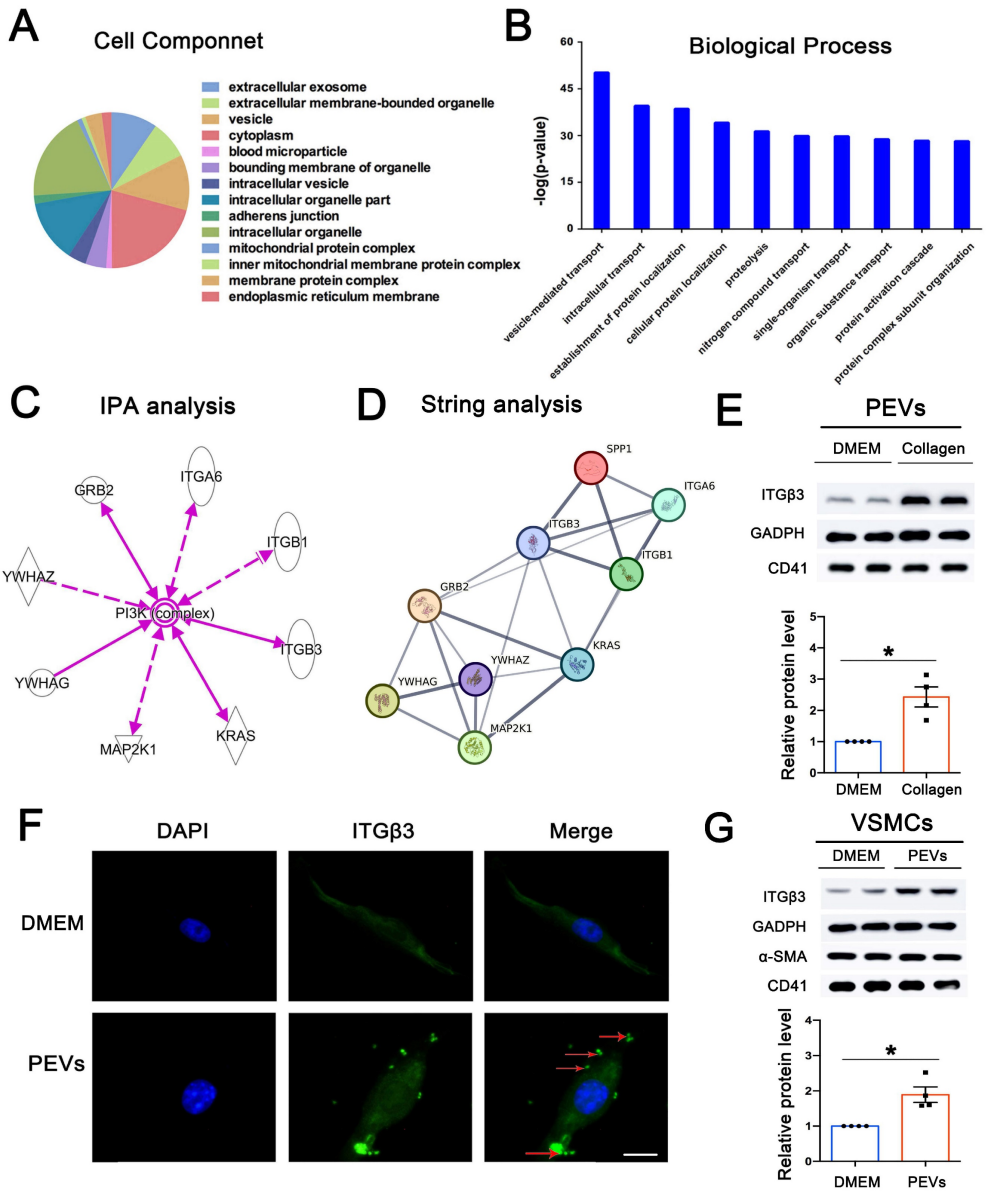
** ITGβ3 on PEVs was the upstream of SPP1. (A)** Cell component analysis of the differentially expressed proteins revealed that these proteins are primarily present in vesicles or extracellular vesicles. (B) Biological process analysis of the differentially expressed proteins ranked vesicle-mediated transport first. (C) IPA was used to predict whether the differentially expressed proteins directly or indirectly activated PI3K-AKT. (D) String analysis predicted that ITGβ3 interacted with SPP1. (E) Collagen was used to activate platelets, and PEVs were generated from the remaining supernatant after platelet removal. Western blotting, used to confirm the proteomics results, showed that compared with the DMEM (control) group, ITGβ3 expression on PEVs was increased in the collagen group. CD41, a marker of PEVs, was also detected by western blotting. (F) Immunofluorescence staining of ITGβ3 (green) and nucleus (blue) of VSMCs after respective treatment with DMEM (control) or PEVs for 1 h. White Arrows indicated the typical regions of dense and punctate ITGβ3 staining. The results revealed that ITGβ3 expressed on PEVs could be delivered to VSMCs after PEV adhesion to VSMCs. Scale bar = 5 μm. (G) Western blotting showed that ITGβ3 expression increased on PEV-treated VSMCs. Both VSMC marker α-SMA and PEV marker CD41 were also detected by western blotting.

To explore candidate proteins expressed on PEVs involved in SPP1-induced FAK/PI3K/AKT phosphorylation, 216 differentially expressed proteins expressed on PEVs were analyzed using GO (Supplementary Table S2). Cell component results revealed the extracellular nature of these proteins, primarily present in vesicles or extracellular vesicles (Figure 5A). In addition, the biological process analysis of the differentially expressed proteins showed that vesicle-mediated transport ranked first (Figure 5B). Moreover, KEGG analysis revealed that the differentially expressed proteins may be involved in PI3K-AKT pathway activation (Figure S4). IPA was then performed to explore the proteins expressed on PEVs involved in PI3K-AKT activation, and 8 proteins, including ITGβ3, ITGβ1, and ITGA6, were predicted to be directly or indirectly associated with PI3K/AKT activation (Figure 5C). Next, string analysis used to analyze whether the candidate proteins interacted with SPP1 showed that these 8 proteins were predicted to directly or indirectly interact with SPP1 (Figure 5D). However, ITGβ3 was the only differentially expressed protein expressed on collagen-induced PEVs compared with the control.

Consistent with proteomics, western blotting results confirmed that compared with that in the DMEM group, ITGβ3 expression was upregulated in PEVs in the collagen group (Figure 5E). Moreover, ITGβ3 was obviously elevated in the PEVs of patients with in-stent restenosis compared with those from healthy volunteers (Figure S5). Therefore, of the candidate proteins, ITGβ3 was chosen for subsequent analysis.

Apart from endocytosis and receptor-mediated binding, extracellular vesicles are able to transfer proteins to the target cells 31, 32. As shown in Figure 5F-G, ITGβ3 expressed on PEVs could be delivered to VSMCs after PEV adhesion, increasing the expression of ITGβ3 on VSMCs. Besides, immunofluorescence staining revealed increased ITGβ3 expression on VSMCs in intimal hyperplasia (Figure S6). Therefore, ITGβ3 was chosen as the candidate upstream protein on PEVs, and subsequent experiments were focused on the role of ITGβ3 in SPP1-mediated PEV adhesion and VSMC dysfunction.

### ITGβ3 on PEVs involved in PEV adhesion and postinjury intimal hyperplasia

**Figure 6:**
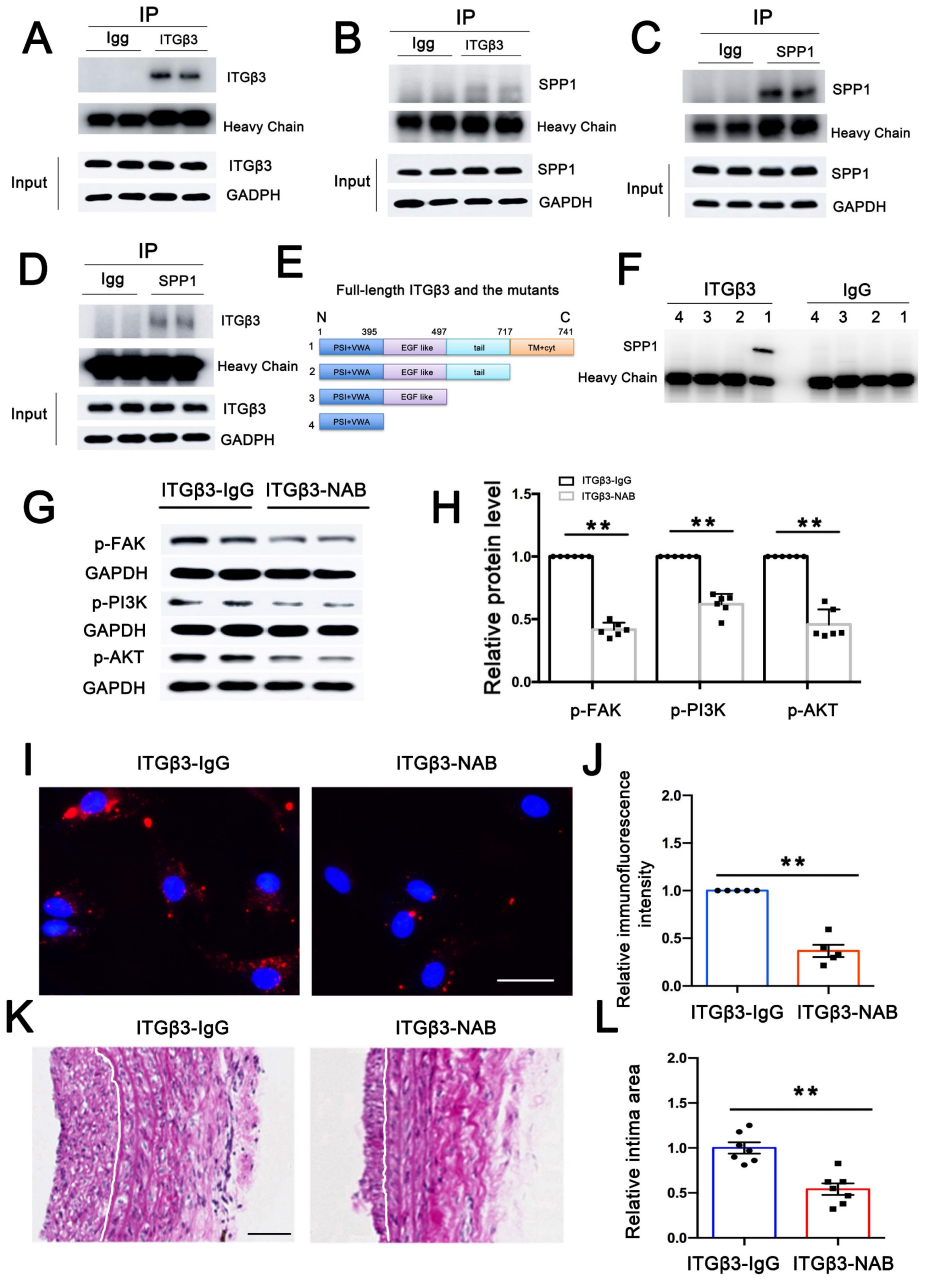
** ITGβ3 on PEVs involved in PEV adhesion and postinjury intimal hyperplasia.** (A-B) Co-IP assay was performed to explore potential interactions between ITGβ3 and SPP1 in adenovirus-transfected VSMCs. VSMC lysate was precipitated, and ITGβ3 enrichment was then evaluated via immunoblotting with ITGβ3 antibody. Co-precipitated SPP1 was also detected via immunoblotting with SPP1 antibody. (C-D) SPP1 enrichment was analyzed by immunoblotting with anti-SPP1 antibody, and the co-precipitated ITGβ3 was assayed with ITGβ3 antibody. The results showed that ITGβ3 and SPP1 were selectively associated in VSMCs. (E) Constructs for the full-length and truncated variants of ITGβ3 were generated. (F) 293T cells were transfected with these plasmids, and Co-IP revealed that SPP1 could only be coprecipitated with the full-length ITGβ3. (G-H) PEVs pretreated with ITGβ3-neutralizing antibody (NAB) or IgG (as a control group) were incubated with VSMCs. Western blotting revealed that ITGβ3-NAB decreased FAK/PI3K/AKT phosphorylation. (I-J) Immunofluorescence assays revealed that the mean immunofluorescence intensity of PEVs adhering to VSMCs was considerably decreased in the ITGβ3-NAB group. Scale bar = 20 μm. (K-L) PEVs pretreated with ITGβ3-NAB or IgG (as a control group) were injected through the rat tail vein. Neointimal hyperplasia was examined using HE staining, and the results revealed that ITGβ3-NAB reduced the neointimal area.

Since ITGβ3 on PEVs could be delivered to VSMCs, co-immunoprecipitation (Co-IP) assay was performed to explore interactions between ITGβ3 and SPP1. As shown in Figures 6A-D, a possibility of close physical interaction between ITGβ3 and SPP1 in VSMCs was observed. To verify the protein interaction domains of ITGβ3, full-length and truncated ITGβ3 mutant variants were constructed (Figure 6E). Subsequently, 293T cells were transfected with these specific plasmids, followed by Co-IP analysis with an anti-ITGβ3 antibody. SPP1 could only be coprecipitated with the full-length ITGβ3 (Figure 6F), suggesting that the transmembrane (TM) and cytoplastic (Cyt) part of ITGβ3 might be critical for its binding to SPP1.

To further investigate the role of ITGβ3 on PEVs and SPP1 in VSMC dysfunction, we used a megakaryoblastic cell line, MEG-01, to produce platelets with specifific ITGβ3 overexpression on PEVs 33, 34. The cells were transfected with adenovirus containing ITGβ3 plasmids or the control plasmids for 48 h and then treated with recombinant human thrombopoietin (rTPO, 100 ng/mL) to produce platelets. Collagen I was used to activate platelets for 1 h and PEVs were subsequently generated from the supernatant. Western blotting confirmed that ITGβ3 was overexpressed on PEVs (Figures S7A-B), which were subsequently used to stimulate VSMCs. CCK-8 assay and scratch wound healing assay were then applied to assess VSMC proliferation and migration, respectively. The results verified that both VSMC migration (Figures S7C-D) and proliferation (Figure S7E) were up-regulated in the ITGβ3 overexpression (ITGβ3-OE) group compared with the control group (ITGβ3-NC).

To investigate whether SPP1 knockdown in VSMC could block the effect of ITGβ3 overexpression PEVs, VSMCs were transfected with SPP1-siRNAs or negative control (SPP1-NC) before ITGβ3 overexpression PEV application. Western blotting confirmed that SPP1 was knockdown in SPP1-siRNAs group (Figures S8A-B). As shown in Figures S8C-E, VSMC proliferation and migration in response to ITGβ3 overexpression PEV application were obviously inhibited in SPP1-siRNA group compared with the SPP1-NC group. Taken together, these results revealed that ITGβ3 overexpression PEVs promote the proliferation and migration of VSMCs, and SPP1 knockdown in VSMCs attenuated the effect of ITGβ3 overexpression PEVs.

Moreover, in order to detect whether ITGβ3 expressed on PEVs was involved in FAK/PI3K/AKT activation, PEVs were pretreated with ITGβ3-neutralizing antibody (NAB) and then incubated with VSMCs. As shown in Figures 6G-H, western blotting revealed that ITGβ3-NAB decreased FAK/PI3K/AKT phosphorylation, indicating that ITGβ3 expressed on PEVs may be involved in FAK/PI3K/AKT phosphorylation. Moreover, the expression level of T-FAK, T-PI3K and T-AKT in ITGβ3-NAB group showed no significant difference with that in the ITGβ3-IgG group (Figure S9).

In addition, the role of ITGβ3 expressed on PEVs in PEV adhesion and postinjury intimal hyperplasia was also assessed. Immunofluorescence assay revealed that the intensity of PEVs that adhered to VSMCs observed in the immunoglobulin G (IgG) group was higher than that measured in the ITGβ3-NAB group (Figure 6I). Meanwhile, PEVs pretreated with ITGβ3-NAB or IgG were injected through the rat tail vein, and the injured carotid artery was collected on day 14 to examine neointimal hyperplasia using HE staining. The results revealed that the area of the neointimal layer was considerably decreased in the ITGβ3-NAB group (Figure 6J). Hence, ITGβ3 on PEVs mediated PEV adhesion and postinjury intimal hyperplasia.

**Figure 7:**
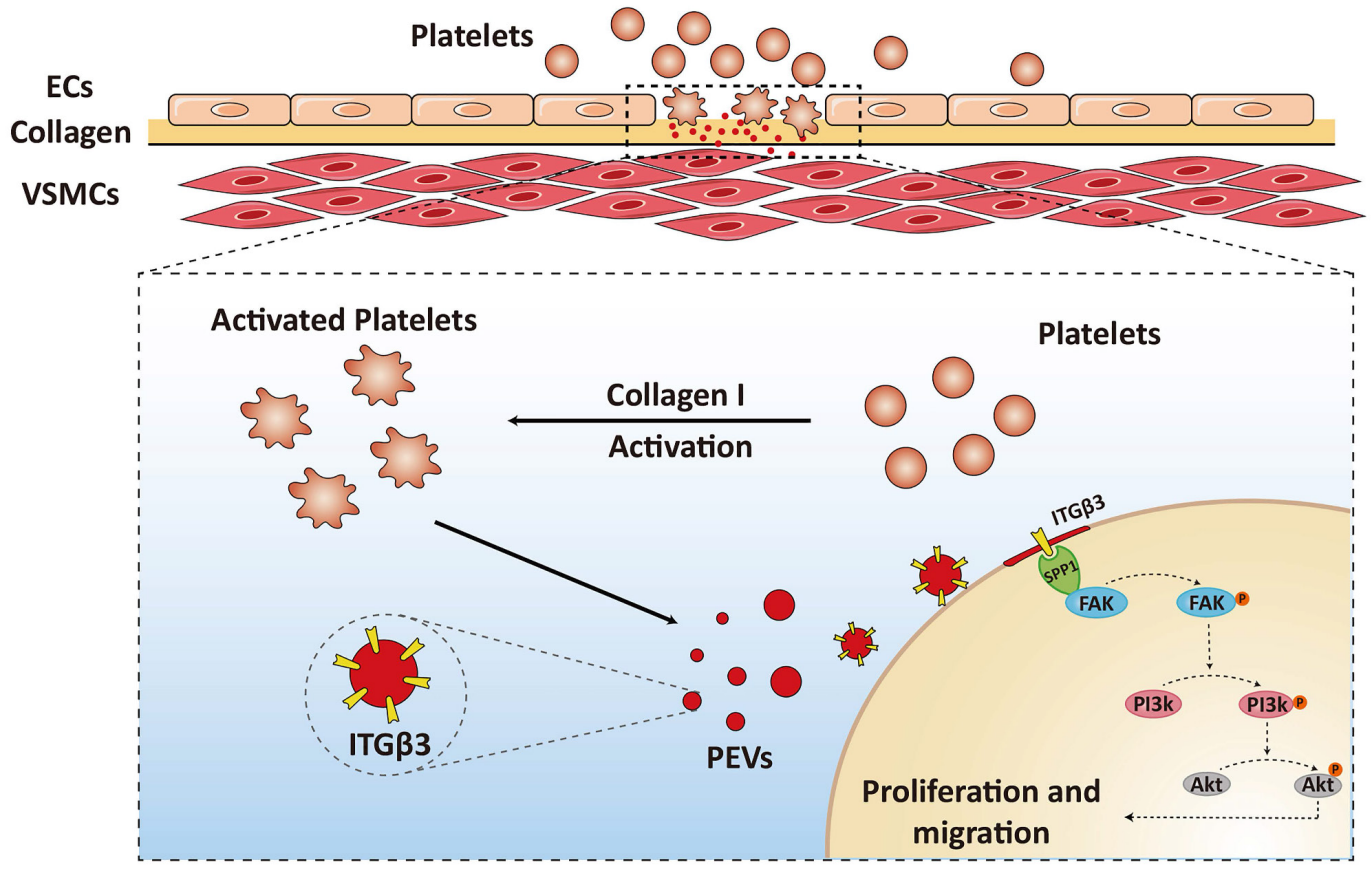
** Schematic drawing of the role of the ITGβ3 expressed on PEVs in VSMC dysfunction.** ITGβ3 on PEVs delivered to VSMCs interacted with SPP1 located in the the cytoplasm of the target cells and activated the downstream of FAK/PI3K/AKT phosphorylation, which facilitated PEV adhesion and postinjury intimal hyperplasia.

Overall, these results suggested that ITGβ3 on PEVs delivered to VSMCs interacted with SPP1 located in the the cytoplasm of the target cells and activated the downstream of FAK/PI3K/AKT phosphorylation, which facilitated PEV adhesion and postinjury intimal hyperplasia (Figure 7).

## Discussion

Following vascular intimal injury or carotid surgery, platelets are immediately recruited and activated at the injured intima, ultimately releasing release bulk PEVs. PEVs released by activated platelets selectively transport abundant genetic materials, including various proteins, lipids, and miRNAs, and mediate the transfer of these contents from platelets to the target cells 14. PEVs have been shown to be involved in the pathogenesis of vascular diseases, and their presence may be a vital predictor of developing thrombosis 14, hypertension 16, or atherosclerosis 35. In the current study, the results revealed that following vascular injury, PEV release triggered by collagen exposure led to enhanced VSMC migration and proliferation, which ultimately promoted intimal hyperplasia (Figure 1). EV adhesion to target cells is currently the most poorly understood step in vesicle-based intercellular communication 20, 21. Therefore, exploring target proteins on PEVs that affect PEV adhesion to VSMCs is crucial for understanding PEV-induced intimal hyperplasia. The present study revealed that ITGβ3 on PEVs serves as the target protein mediating this process.

Integrins, cell surface receptors, play diverse roles in mediating cellular adhesion and homeostasis 36. Research has indicated that EVs enriched with integrin β1 can enhance monocyte adhesion and contribute to liver inflammation in nonalcoholic steatohepatitis 37. In addition, ITGβ3 has been reported to serve as a mediator of EV adhesion by interacting with heparan sulfate proteoglycans in the progression of breast cancer 38. Moreover, ITGβ3 on PEVs was shown to mediate platelet-nasopharyngeal carcinoma cell communication, thereby promoting its distant metastasis 39. However, whether integrins on PEVs are involved in intimal hyperplasia remains unclear. In the present study, increased ITGβ3 expression was observed on pathological PEVs, which promoted PEV adhesion to VSMCs and mediated platelet-VSMC communication, ultimately facilitating postinjury intimal hyperplasia (Figure 6I-L). Moreover, ITGβ3 was upregulated in the PEVs of patients with in-stent restenosis compared with those from healthy volunteers (Figure S3). Overall, the expression of ITGβ3 on PEVs was positively correlated with the progression of intimal hyperplasia.

Besides, one strength of the present study is that scRNA-seq was performed, a technique that enables both the unbiased and comprehensive characterization of cell subpopulation for phenotyping and quantification of relative gene abundance within control or complex diseased tissues in various vascular diseases 40, 41. However, the limitations of scRNA-seq should be acknowledged and carefully considered when analyzing such datasets. For example, scRNA-seq can only reflect variations at the transcript level, not protein level, and single-cell heterogeneity may appear artificially increased 42. To overcome such limitations, a combination of scRNA-seq and bulk proteomics was used to elucidate the mechanism of PEV-induced intima hyperplasia.

Based on the bulk proteomic analysis, 129 differentially expressed proteins were identified in PEV-treated VSMCs (Supplementary Table S1), with the ability to participate in focal adhesion and PI3K-Akt signaling, consistent with the findings of the SPP1^high^ VSMC subpopulation. Of note, SPP1 interacts with integrin involved in cell adhesion 30, 43 and is known to participate in vascular diseases including, but not limited to, vascular calcification 44, 45, atherosclerosis 46, 47, and intimal hyperplasia 48. In response to intimal hyperplasia, PEV adhesion to VSMCs is critical for PEV-induced VSMC dysfunction and intimal hyperplasia. However, it was not known whether SPP1 regulates PEV adhesion to VSMCs. The present study confirmed that increased SPP1 expression positively promoted PEV adhesion to VSMCs and drove VSMC dysfunction (Figure 4). Besides, SPP1 contains an Arg-Gly-Asp (RGD) binding domain that enables ITGβ3 binding 49, and the present study showed that ITGβ3 could selectively interact with SPP1 in PEV-treated VSMCs (Figures 6E-F) to facilitate PEV adhesion to VSMCs during intimal hyperplasia. These processes represent a previously unexplored mechanism through which ITGβ3-SPP1 interaction regulates PEV adhesion in intimal hyperplasia.

As FAK is an essential kinase in the focal adhesion pathway and upstream of PI3K/AKT activation 50, 51, FAK/PI3K/AKT phosphorylation in PEV-treated VSMCs was analyzed. The results verified that FAK inhibitor not only decreased FAK/PI3K/AKT phosphorylation but also attenuated PEV-induced VSMC migration and proliferation compared with the control group (Supplementary Figure S10), indicating that FAK/PI3K/AKT phosphorylation is involved in PEV-induced VSMC dysfunction. In addition, both ITGβ3 expressed on PEVs (Figures 6G-H) and SPP1 in VSMCs (Figures 4A-B) were found essential for FAK/PI3K/AKT phosphorylation in PEV-treated VSMCs. Thus, FAK/PI3K/AKT phosphorylation may be a critical factor for ITGβ3-SPP1 regulating PEV-induced VSMC dysfunction.

Although the administration of antiplatelet agents can decrease the occurrence of carotid artery stenosis by decreasing platelet aggregation at the site of injured vascular 52, 53, in-stent restenosis remains a major challenge. A previous study reported that an antiplatelet agent such as acetylsalicylic acid has no effect on microvesicles, including PEVs 54. Therefore, exploring potential targets on PEVs may be crucial to develop clinical strategies for preventing intimal restenosis. Based on the present study, an ITGβ3 neutralizing antibody was used to block ITGβ3 expression on PEVs *in vitro*, which were then injected into an animal model of vascular injury *in vivo*. This blockade attenuated PEV-induced in-stent hyperplasia (Figures 6K-L). Besides, an injectable nanogel was developed to provide a sustained release of siRNA-ITGβ3 (RGD-Nanogel/siRNA-ITGβ3), which blocked SPP1-ITGβ3 interaction in infrapatellar fat pad and attenuated osteoarthritis 49. Hence, targeting the ITGβ3-SPP1 interaction may have potential clinical implications for preventing intimal hyperplasia. However, the role of PEVs activated at the injured intimal site in vascular remodeling is extremely complex. A study demonstrated that activated PEVs delivered TGF-β1 to endothelial progenitor cells, promoting their proliferation and vascular EC repair, suggesting that PEVs act as a potential therapeutic target for vascular injury 33. Additional studies on the potential mechanisms by which PEVs regulate vascular remodeling may shed light on in-stent restenosis.

Taken together, the current study revealed an essential and previously unknown function of SPP1 in PEV adhesion to VSMCs, which promotes postinjury intimal hyperplasia. The function of SPP1 in this capacity is dependent on its interactions with ITGβ3, whose expression is increased on pathological PEVs, leading to the activation of the FAK/PI3K/Akt axis in VSMCs. Therefore, SPP1-mediated interactions with ITGβ3 on PEVs represents a novel mechanism driving intimal hyperplasia, which may be a process critical for vascular metastasis. Finally, increased ITGβ3 expression on PEVs may be a potential clinical biomarker to predict intimal hyperplasia after vascular injury.

## Supplementary Material

Supplementary figures and tables.

## Abbreviations

VSMC: vascular smooth muscle cell
PEVs: platelet-derived extracellular vesicles
SPP1: secreted phosphoprotein 1
FAK: focal adhesion kinase
PI3K: phosphoinositide 3 kinase
Akt: protein kinase B
ITGβ3: integrin beta 3
Co-IP: Co-immunoprecipitation
ECs: endothelial cells
HE: hematoxylin and eosin
PBS: phosphate-buffered saline
DAPI: 4, 6-diamidino-2-phenylindole
DMEM: Dulbecco's Modified Eagle Medium
FBS: fetal bovine serum
HRP: horseradish peroxidase
ECL: chemiluminescence
α-SMA: α-smooth muscle actin
scRNA-seq: single-cell RNA Sequenceing
UMAP: uniform manifold approximation and projection
col1a1: collagen alpha-1(I) chain
KEGG: kyoto encyclopedia of genes and genome
MF: molecular function
siRNA: small interfering RNA
GO: gene ontology
IPA: ingenuity pathways analysis
NAB: neutralizing antibody
lgG: immunoglobulin G
RGD: Arg-Gly-Asp
