# Supplementary material for: Integrin β3-mediated platelet extracellular vesicle adhesion facilitates vascular smooth muscle cell dysfunction in postinjury intimal hyperplasia
Source: Int J Biol Sci. 2025 Mar 3;21(6):2380–95. doi: 10.7150/ijbs.101391 (PMC12035895; doi:10.7150/ijbs.101391)
Supplement: Supplementary file 1 — Supplementary figures and tables. [file ijbsv21p2380s1.pdf]

Integrin β3-enriched platelet extracellular vesicles interaction with SPP1 in VSMCs facilitate postinjury intimal hyperplasia

Fei Zhuang[1#], Zhi-tong Liu[1#], Guo Zhou[1], Feng Liang[1], Ying-hua Wang[1], Long Chen[1], Wei-feng Zhang[1], Ling-hong Shen[1], Yan-qiao Lu[1], huan-huan huo[1], Xin Shi[1⊠], Liang fang[2⊠], Ben He[1⊠].

1. Department of Cardiology, Shanghai Chest Hospital, School of Medicine, Shanghai Jiao Tong University, Shanghai 200030, China.

2. Department of Cardiac Surgery, Shanghai Chest Hospital, Shanghai Jiao Tong University School of Medicine, Shanghai 200030, China.

#Contributed equally.

*email: heben@sjtu.edu.cn; fangliang0@foxmail.com; iskysx@163.com;

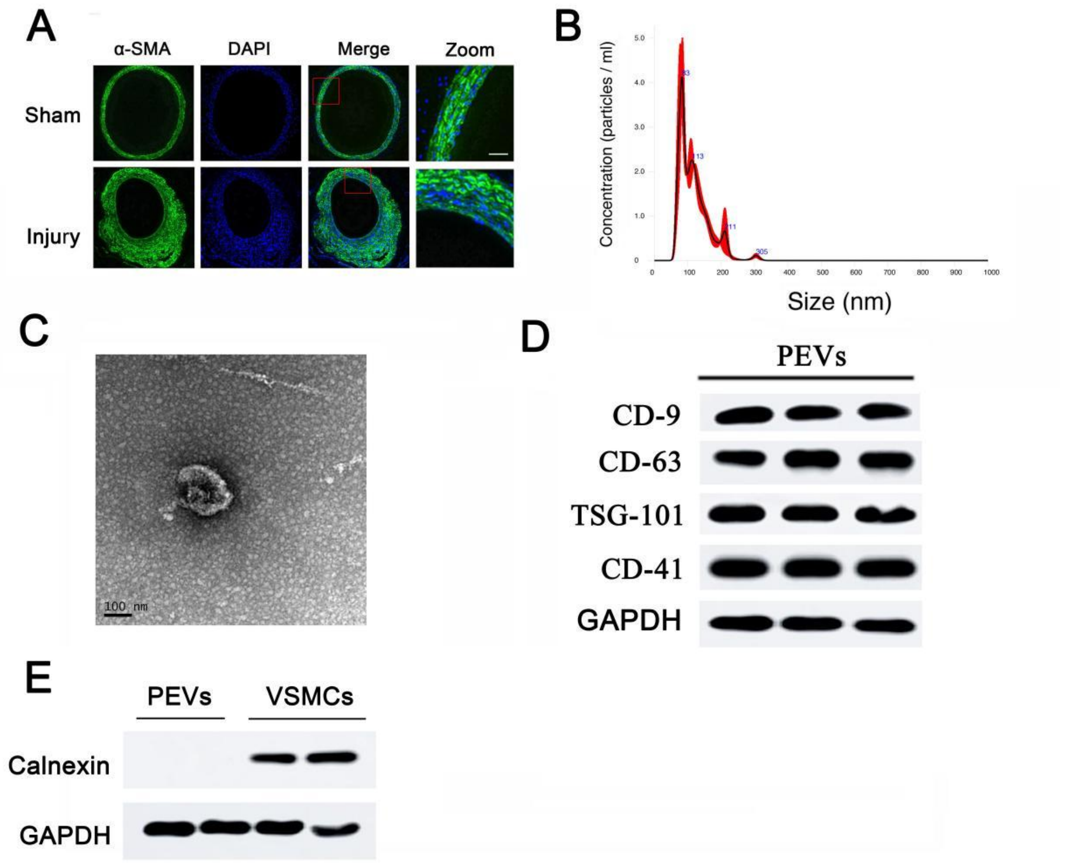

Figure S1. Characterize the diameter and markers of the isolated PEVs. (A) Immunofluorescence staining showed that intimal hyperplasia mainly comprised VSMCs after intimal injury. VSMCs were stained with α-SMA (green) and their

nucleus were stained with DAPI (blue). Scale bar = 50 μm. (B) The diameter of PEVs was assessed by NTA, and the results showed that there were 4 peaks at 83 nm, 113 nm, 211nm and 305 nm. (C) PEV morphology and size were also evaluated by Electron microscope. (D) Western blotting results indicated both the markers of EVs (CD9, CD63 and TSG-101) and platelet-derived EV specific marker CD41 were expressed on the extracted samples. (E) Calnexin, expressed in the endoplasmic reticulum of cells not in EVs, was used as the negative control of the extracted PEVs. The expression of calnexin in PEVs and VSMCs was detected by western blotting respectively.

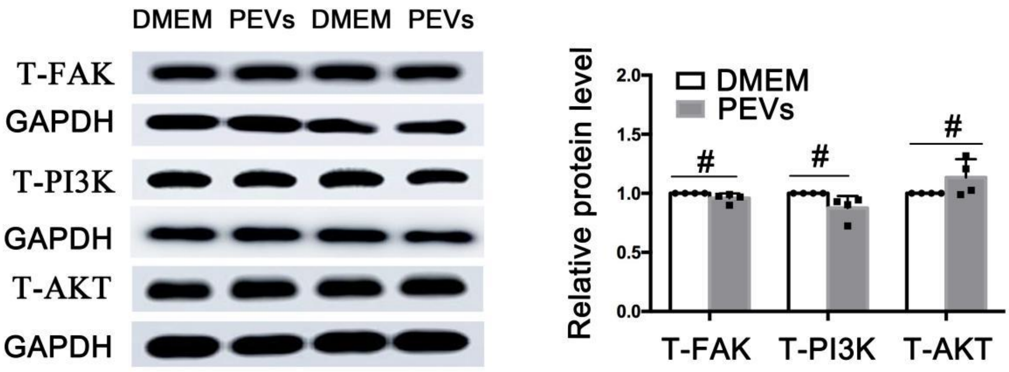

Figure S2. Western blotting analysis of total FAK (T-FAK), total PI3K (T-PI3K) and total AKT (T-AKT) expression level in PEV-treated VSMCs. The values are shown as mean ± SD. # P ⩾0.05.

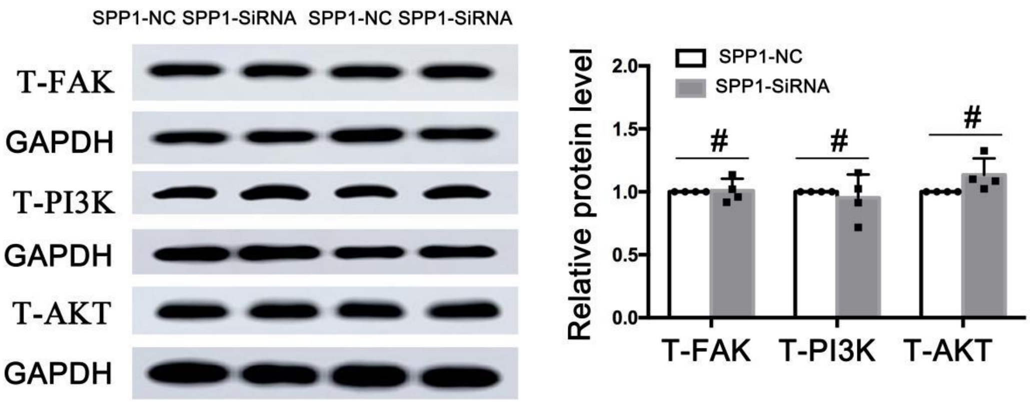

Figure S3. The expression level of T-FAK, T-PI3K, and T-AKT in SPP1-siRNA group showed no significant difference with that in the SPP1-NC group. The values are shown as mean ± SD. # P ⩾0.05.

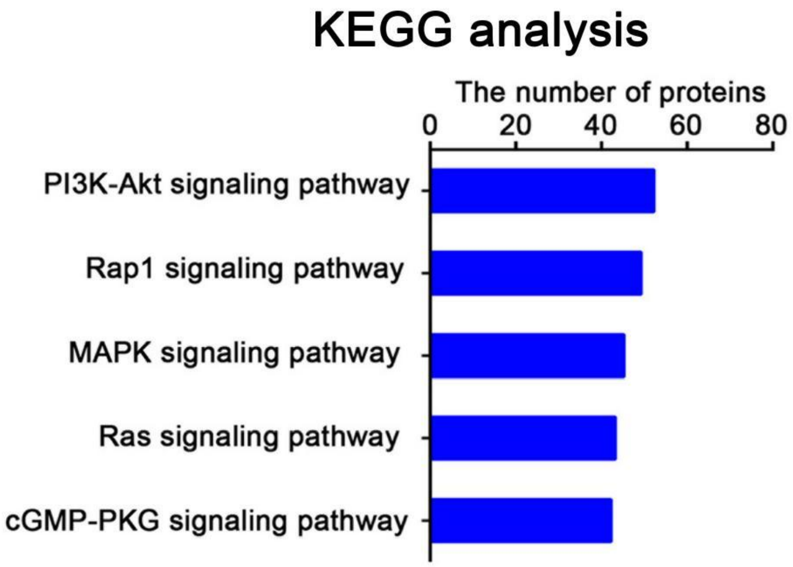

Figure S4. KEGG analysis of the differentially expressed proteins in collagen-induced PEVs.

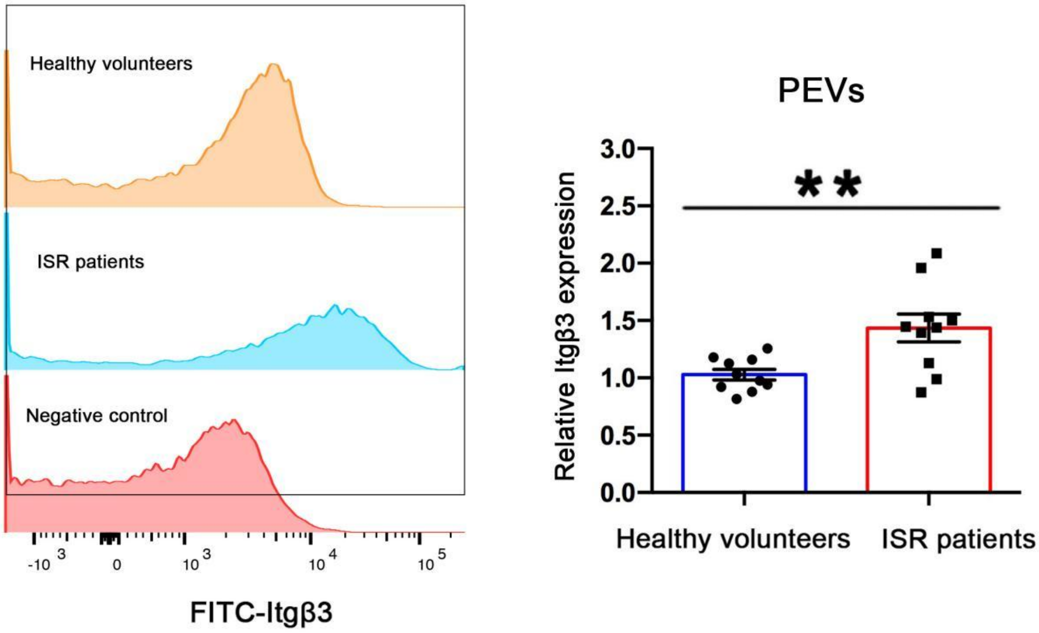

Figure S5. The expression of ITGβ3 was obviously elevated on PEVs of in-stent restenosis (ISR) patients compared with those from healthy volunteers (n=10). (A-B) Flow cytometry analysis was used to assessed the expression of ITGβ3 on PEVs of in-stent restenosis (ISR) patients compared with those from healthy volunteers. The values are shown as mean ± SD. * $P < 0.05$, ** $P < 0.01$.

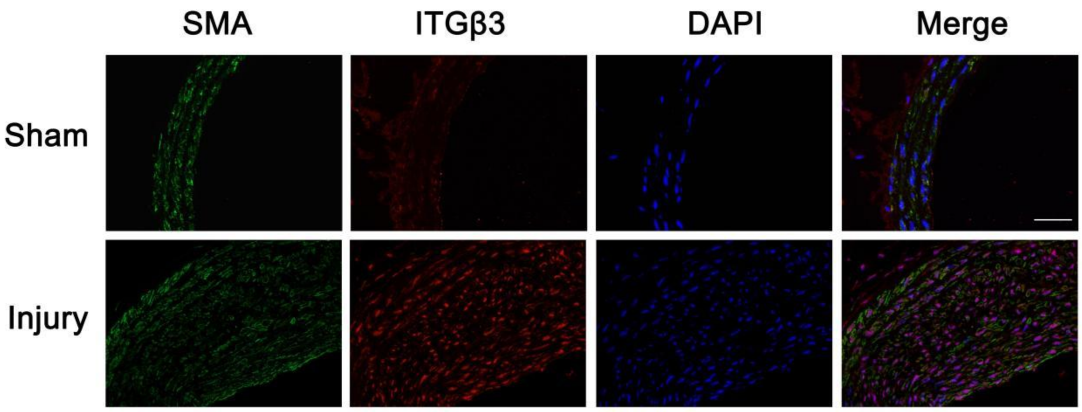

Figure S6. Immunofluorescence analysis revealed the increased ITGβ3 expression in VSMC in intimal hyperplasia.

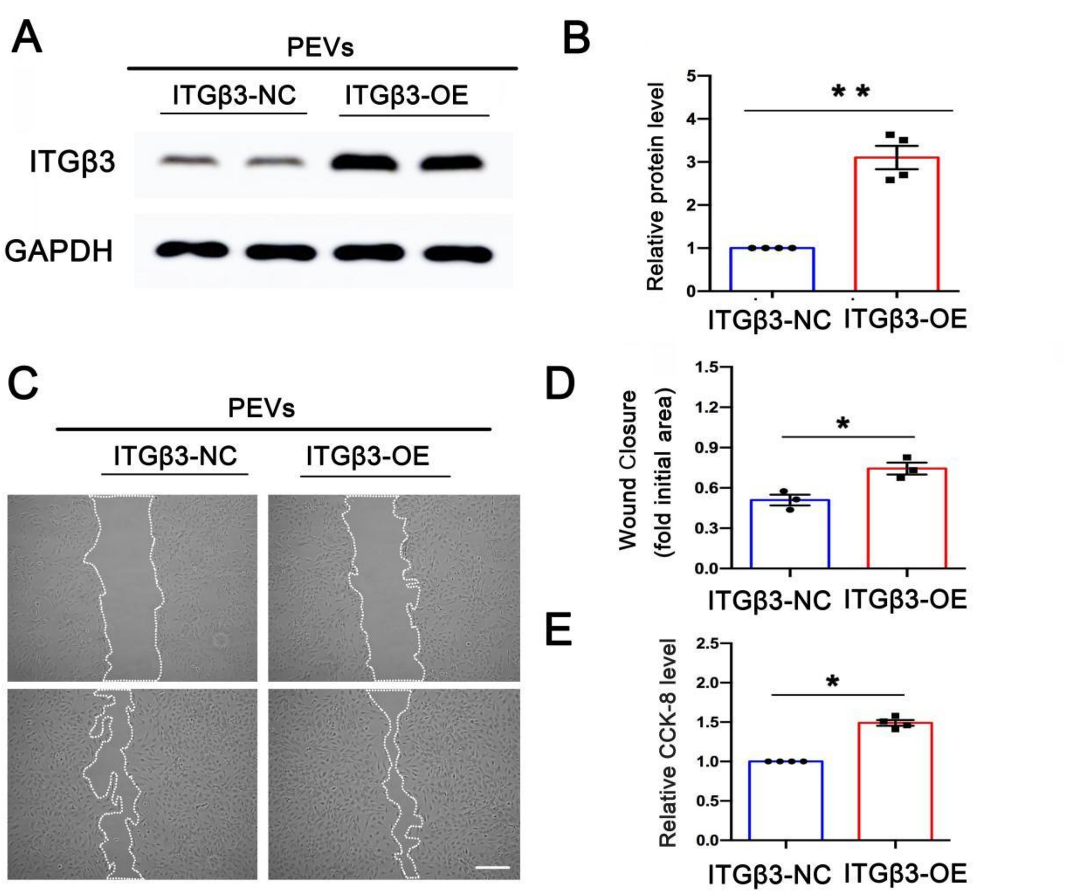

Figure S7. ITGβ3 overexpression PEVs promoted the proliferation and migration of VSMCs. (A-B) MEG-01 were transfected with adenovirus containing ITGβ3 overexpression plasmids (ITGβ3-OE) or the control plasmids (ITGβ3-NC) for 48 h and then treated with recombinant human thrombopoietin (rTPO, 100 ng/mL) to produce platelets. Collagen I was used to activate platelets for 1 h and PEVs were subsequently generated from the supernatant. The expression of ITGβ3 on PEVs was then assessed by western blotting. (C-D) The migration of VSMCs in DMSO group or FAK inhibitor group was assessed by scratch wound healing analysis. Scale bar=200 μm. (D) CCK-8 was used to analyze VSMC proliferation. The values are shown as mean ± SD. * $P < 0.05$, ** $P < 0.01$.

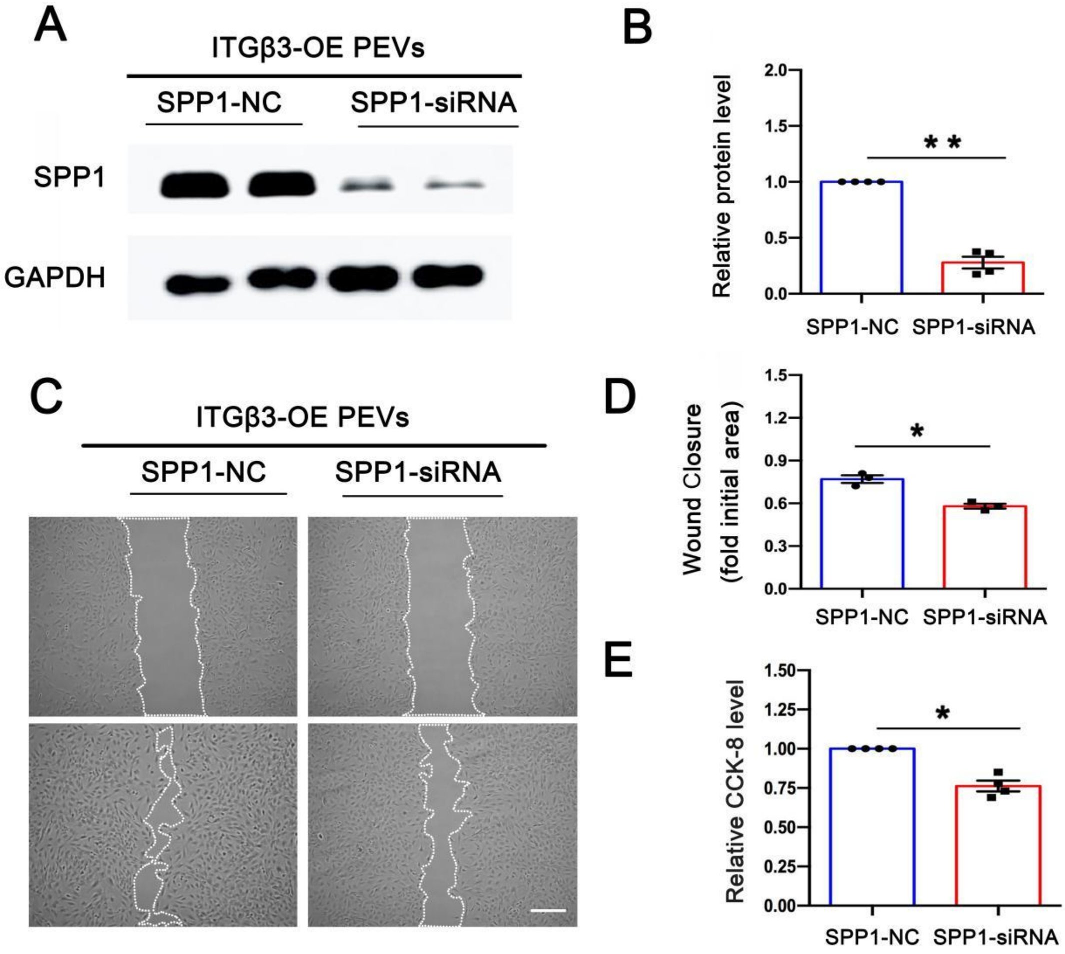

Figure S8. SPP1 knockdown in VSMC attenuated the effect of ITGβ3 overexpression PEVs. (A-B) VSMCs were transfected with SPP1-siRNA or SPP1-NC for 48 h and SPP1 expression was then assessed by western blotting. (C-D) After that, VSMCs were treated with ITGβ3-OE PEVs, and the migration of VSMCs in SPP1-siRNA group or SPP1-NC group was assessed by scratch wound healing analysis. Scale bar=200 μm. (D) CCK-8 was used to analyze VSMC proliferation. The values are shown as mean ± SD. * $P < 0.05$, ** $P < 0.01$.

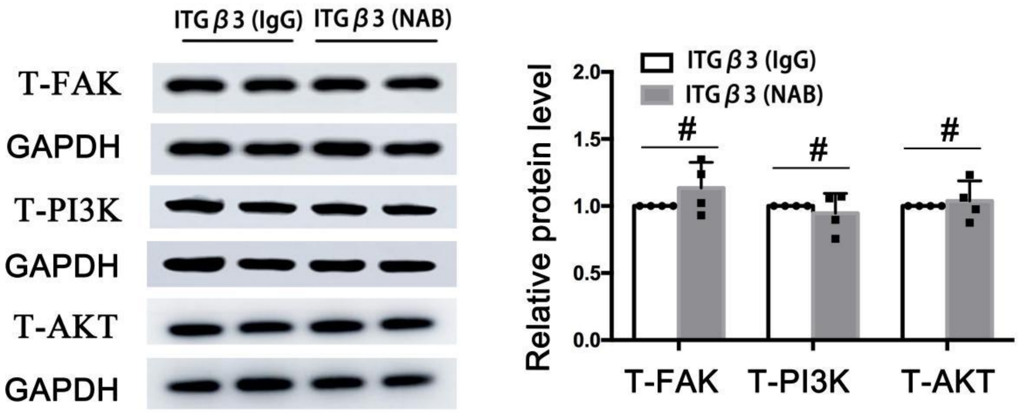

Figure S9. The expression level of T-FAK, T-PI3K, and T-AKT in ITGβ3-NAB group showed no significant difference with that in the ITGβ3-IgG group. The values are shown as mean ± SD. # $P \geq 0.05$.

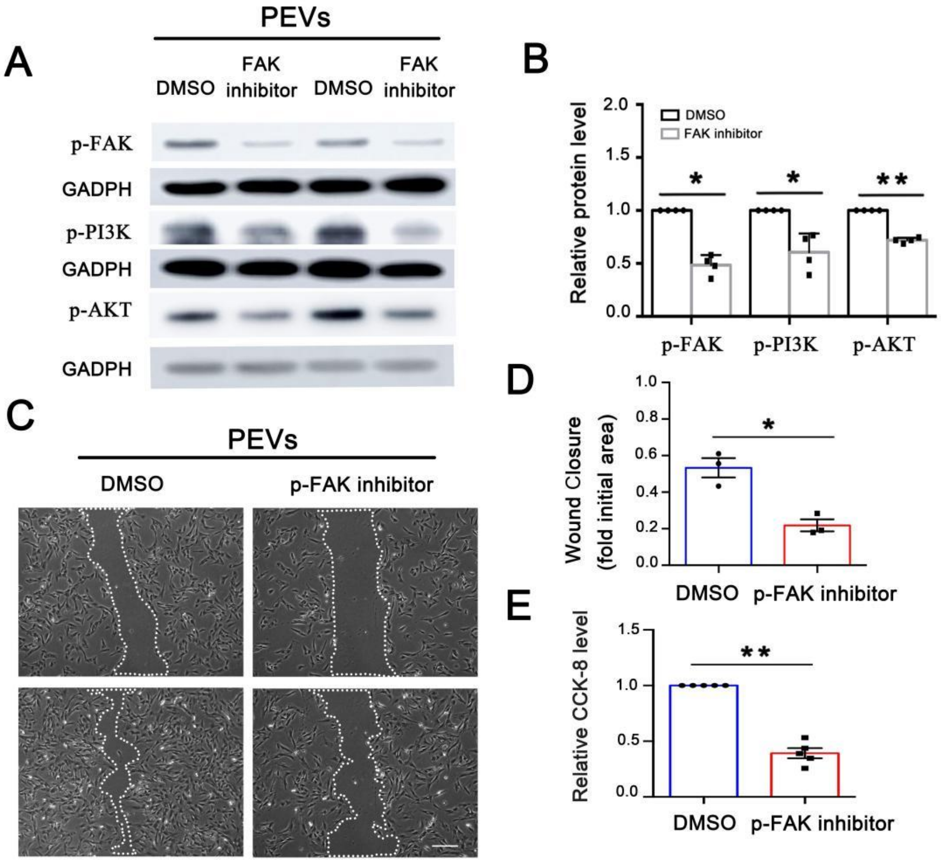

Figure S10. FAK inhibitor inhibited PEVs-induced FAK/PI3K/AKT phosphorylation and VSMC dysfunction. (A-B) FAK inhibitor and DMSO (the

control group) were used to pretreat with VSMCs respectively before PEV application. The expression of p-FAK, p-PI3K and p-AKT in VSMCs were then assessed by western blottling. (C-D) The migration of VSMCs in response to DMSO and FAK inhibitor was also detected by scratch wound healing analysis. Scale bar=200 μm. (D) CCK-8 was used to analyze VSMC proliferation. The values are shown as mean ± SD. * P < 0.05, ** P < 0.01.

Tabel S1 129 proteins showed significant differences between PEV-treated VSMCs and control groups (P-value < 0.05 and fold change > 2).

Protein	Protein Description	Gene Name	ratio（PEV/Control）	p-value
ENSRNOP00000045757	APC regulator of WNT signaling pathway 2	Apc2	10.43338259	0.002235387
ENSRNOP00000093639	epiplakin 1	Eppk1	8.997361815	0.007912259
ENSRNOP00000071872	oxidized low density lipoprotein receptor 1	Olr1	5.569995106	0.000598324
ENSRNOP00000067271	integrin subunit beta 3	Itgb3	4.219715978	0.002312709
ENSRNOP00000025718	notch receptor 2	Notch2	4.114215629	0.000655875
ENSRNOP00000020108	interleukin 1 receptor-like 1	Il1rl1	3.910183567	0.005208203
ENSRNOP00000077964	LRR binding FLII interacting protein 1	Lrrfip1	3.329791404	0.026076931
ENSRNOP00000072517	MICAL-like 2	Micall2	3.299539986	0.018803354
ENSRNOP00000023139	protein phosphatase 2, regulatory subunit B, delta	Ppp2r2d	3.124028954	0.013802083
ENSRNOP00000074084	laminin subunit alpha 5	Lama5	3.073467051	3.06746E-06
ENSRNOP00000004713	ADAM metallopeptidase with thrombospondin type 1 motif, 4	Adamts4	2.752527974	0.030665927
ENSRNOP00000029969	dystrophin	Dmd	2.740075785	0.013785878
ENSRNOP00000071884	tenascin C	Tnc	2.682919001	0.001321491
ENSRNOP0	ring finger protein 149	Rnf149	2.569338044	0.0301

0000018684				96317
ENSRNOP0 0000011298	solute carrier family 2 member 3	Slc2a3	2.482269976	0.0008 86343
ENSRNOP0 0000028074	cleavage and polyadenylation specific factor 7	Cpsf7	2.420726089	0.0311 3781
ENSRNOP0 0000022432	ubiquitin specific peptidase 10	Usp10	2.322054484	0.0037 68495
ENSRNOP0 0000010806	DEAD-box helicase 27	Ddx27	2.272017875	0.0091 57339
ENSRNOP0 0000011904	cellular communication network factor 3	Ccn3	2.193106802	0.0119 25593
ENSRNOP0 0000062358	secreted phosphoprotein 1	Spp1	2.099047677	0.0067 31151
ENSRNOP0 0000028288	elongator acetyltransferase complex subunit 6	Elp6	2.077952211	0.0172 27558
ENSRNOP0 0000001916	serpin family E member 1	Serpin e1	2.067643449	0.0012 92639
ENSRNOP0 0000001383	tumor protein, translationally-controlled 1	Tpt1	2.05152988	0.0214 85566
ENSRNOP0 0000051976	cleavage stimulation factor subunit 1	Cstf1	2.002821541	0.0214 93598
ENSRNOP0 0000009008	sulfatase modifying factor 1	Sumf1	2.000249558	0.0068 13432
ENSRNOP0 0000025634	MOB kinase activator 3A	Mob3a	1.949048998	0.0176 38587
ENSRNOP0 0000035354	RFT1 homolog	Rft1	1.926084187	0.0148 03719
ENSRNOP0 0000069322	thrombospondin 1	Thbs1	1.85452317	0.0022 88111
ENSRNOP0 0000024286	mitochondrial ribosomal protein L48	Mrpl48	1.851059764	0.0340 70505
ENSRNOP0 0000000737	laminin subunit alpha 4	Lama4	1.850801802	0.0073 82754
ENSRNOP0 0000009467	pregnancy-zone protein	Pzp	1.819284056	0.0388 78025
ENSRNOP0 0000019880	interleukin 1 receptor-like 2	Il1rl2	1.817738629	0.0073 49802
ENSRNOP0 0000007577	matrix Gla protein	Mgp	1.816616444	0.0398 48558
ENSRNOP0 0000017900	iron responsive element binding protein 2	Ireb2	1.812552684	0.0033 37292
ENSRNOP0 0000000080	down-regulator of transcription 1	Dr1	1.812217904	0.0171 39557
ENSRNOP0	solute carrier family 4 member	Slc4a7	1.797419146	0.0126

0000008759	7			25725
ENSRNOP0	filamin A interacting protein			0.0004
0000045927	1-like	Filip1l	1.794526239	36484
ENSRNOP0	N(alpha)-acetyltransferase 35,			0.0447
0000071899	NatC auxiliary subunit	Naa35	1.784453543	947
ENSRNOP0	protein phosphatase 1,	Ppp1r1		0.0023
0000050977	regulatory subunit 13 like	3l	1.783906099	90327
ENSRNOP0	DnaJ heat shock protein			0.0173
0000017740	family (Hsp40) member C8	Dnajc8	1.781995608	48918
ENSRNOP0				0.0201
0000006554	LIM domain containing 1	Limd1	1.754586969	88364
ENSRNOP0				0.0420
0000088195	actin, beta	Actb	1.735623248	67654
ENSRNOP0	protein tyrosine phosphatase,			0.0006
0000023831	non-receptor type 9	Ptpn9	1.729498708	02265
ENSRNOP0		Vps37		0.0382
0000001445	VPS37B subunit of ESCRT-I	b	1.726230566	86112
ENSRNOP0	transmembrane p24 trafficking			0.0006
0000006802	protein 4	Tmed4	1.721524962	36937
ENSRNOP0	basophilic leukemia expressed			0.0073
0000027869	protein BLES03	Bles03	1.679001378	18039
ENSRNOP0				0.0428
0000075193	caspase 7	Casp7	1.66614971	07649
ENSRNOP0				0.0004
0000042691	aggrecan	Acan	1.65999968	17206
ENSRNOP0		Gask1		0.0246
0000013525	golgi associated kinase 1B	b	1.62111634	9549
ENSRNOP0	C-type lectin domain family 2,			0.0291
0000069630	member G	Clec2g	1.616221529	44434
ENSRNOP0	transforming growth factor,			0.0003
0000013516	beta 3	Tgfb3	1.604515327	34315
ENSRNOP0				0.0281
0000019162	ribosomal protein L35	Rpl35	1.600683947	07502
ENSRNOP0	endothelin converting enzyme			0.0040
0000062928	1	Ece1	1.579592674	55207
ENSRNOP0				0.0135
0000033144	ribosomal protein s25	Rps25	1.573775112	70495
	abhydrolase domain			
ENSRNOP0	containing 6, acylglycerol			0.0306
0000012271	lipase	Abhd6	1.573219471	35704
ENSRNOP0				0.0038
0000039985	ubiquitin specific peptidase 13	Usp13	1.57241862	6722
ENSRNOP0	cellular communication			0.0009
0000019501	network factor 1	Ccn1	1.568027288	32457

ENSRNOP00000009260	TANK-binding kinase 1	Tbk1	1.566040873	0.002016367
ENSRNOP00000013496	low density lipoprotein receptor	Ldlr	1.55883152	0.000897319
ENSRNOP00000006880	integrin subunit alpha 4	Itga4	1.558079304	0.012270323
ENSRNOP00000069382	solute carrier organic anion transporter family, member 2a1	Slco2a1	1.557521937	0.013707635
ENSRNOP00000007276	zinc finger CCCH-type containing 15	Zc3h15	1.538857148	0.049705981
ENSRNOP00000014211	nucleolar protein 6	Nol6	1.522121523	0.02425592
ENSRNOP00000043782	methylsterol monooxygenase 1	Msmo1	1.521915446	0.007102719
ENSRNOP00000020520	leucine rich repeat containing 32	Lrrc32	1.515086606	0.023947274
ENSRNOP00000066613			1.514296807	0.003664423
ENSRNOP00000010762	Bcl2-like 1	Bcl2l1	1.509324755	0.010130335
ENSRNOP00000042417	leucine rich repeats and calponin homology domain containing 3	Lrch3	1.503211463	0.019532523
ENSRNOP00000001695	collagen type VI alpha 2 chain	Col6a2	0.664863168	0.012560362
ENSRNOP00000036154	frizzled class receptor 2	Fzd2	0.660014929	0.008884294
ENSRNOP00000037346	SMAD family member 3	Smad3	0.657480982	0.030305728
ENSRNOP00000054231	mannosidase, alpha, class 1A, member 1	Man1a1	0.657007664	0.035407778
ENSRNOP00000004955	fibronectin leucine rich transmembrane protein 2	Flrt2	0.65696301	0.032044814
ENSRNOP00000072765	mannosidase beta	Manba	0.65693694	0.008817508
ENSRNOP00000047275	WD repeat domain 44	Wdr44	0.656626103	0.006579017
ENSRNOP00000010811	N-myc downstream regulated 1	Ndrg1	0.648416203	0.003031769
ENSRNOP00000003251	A-kinase anchoring protein 1	Akap1	0.647436272	0.032367711
ENSRNOP00000021716	coenzyme Q9	Coq9	0.644291834	0.018131186

ENSRNOP00000077937	integrin subunit beta 5	Itgb5	0.640369843	0.001564715
ENSRNOP00000025966	natriuretic peptide receptor 3	Npr3	0.639560881	0.004369462
ENSRNOP00000055628	non-SMC condensin I complex, subunit G	Ncapg	0.639340699	0.014242701
ENSRNOP00000022726	eukaryotic elongation factor-2 kinase	Eef2k	0.634931816	0.027654024
ENSRNOP00000010127	C1q and TNF related 5	C1qtnf5	0.634865525	0.000324111
ENSRNOP00000015821	ring finger protein 7	Rnf7	0.634594395	0.000374815
ENSRNOP00000043878	DAB adaptor protein 2	Dab2	0.628060515	0.009908881
ENSRNOP00000017045	regulator of MON1-CCZ1	Rmc1	0.627162112	0.007964316
ENSRNOP00000063448	VPS41 subunit of HOPS complex	Vps41	0.626628803	0.026078174
ENSRNOP00000034162	serine threonine kinase 39	Stk39	0.624796947	0.024603846
ENSRNOP00000019579	insulin receptor substrate 1	Irs1	0.62310402	0.022522386
ENSRNOP00000075631	gelsolin	Gsn	0.609084021	0.037843942
ENSRNOP00000065711	tubulin, beta 1 class VI	Tubb1	0.60460115	0.027292522
ENSRNOP00000017600	adducin 3	Add3	0.589621872	0.002964914
ENSRNOP00000044578	membrane metallo-endopeptidase	Mme	0.579103509	0.038168636
ENSRNOP00000071870	spermidine/spermine N1-acetyltransferase family member 2	Sat2	0.578899599	0.047573617
ENSRNOP00000072431	kinesin family member 1B	Pgd	0.578076374	0.017276799
ENSRNOP00000029705	CDKN2A interacting protein	Cdkn2aip	0.573612434	0.009472546
ENSRNOP00000008882	ankyrin repeat domain 24	Ankrd24	0.567607394	0.000982094
ENSRNOP00000044213			0.567498428	0.034245664
ENSRNOP00000073048	keratin 78	Krt78	0.565741753	0.029785939
ENSRNOP0	colony stimulating factor 1	Csf1	0.54902939	0.0232

0000073134				50381
ENSRNOP00000051391	family with sequence similarity 172, member A	Fam172a	0.545053423	0.031283608
ENSRNOP00000039338	thiosulfate sulfurtransferase	Tst	0.54063301	0.017981977
ENSRNOP00000013606	zinc finger, C3HC-type containing 1	Zc3hc1	0.536023548	0.032092409
ENSRNOP00000021646	hydroxysteroid (17-beta) dehydrogenase 4	Hsd17b4	0.531428828	0.000744201
ENSRNOP00000006284	trans-L-3-hydroxyproline dehydratase	L3hypdh	0.520010586	0.025106346
ENSRNOP00000035225	TNF superfamily member 18	Tnfsf18	0.513117548	0.001634322
ENSRNOP00000014346	cellular communication network factor 5	Ccn5	0.511410011	0.001950875
ENSRNOP00000064117	zinc finger protein 326	Zfp326	0.505966569	0.005987598
ENSRNOP00000047323	cyclin Y-like 1	Ccnyl1	0.50409236	0.012395146
ENSRNOP00000034042	mitochondrial ribosomal protein L24	mrpl24	0.499675522	0.013311626
ENSRNOP00000012109	carboxypeptidase Z	Cpz	0.494167842	0.013252469
ENSRNOP00000013851	RAB30, member RAS oncogene family	Rab30	0.487750578	0.011092717
ENSRNOP00000017065	complement C1q C chain	C1qc	0.468250427	0.002670482
ENSRNOP00000020770	lon peptidase 2, peroxisomal	Lonp2	0.466118852	0.039425713
ENSRNOP00000012092	amyloid P component, serum	Apcs	0.460874189	0.002605655
ENSRNOP00000022963	phosphoglucomutase 2-like 1	Pgm2l1	0.439364886	0.018608891
ENSRNOP00000020532	osteoglycin	NEWGENE_1308171	0.433381996	0.001298053
ENSRNOP00000038351	carbonyl reductase 4	Cbr4	0.429292702	0.021155599
ENSRNOP00000013994	malonyl-CoA-acyl carrier protein transacylase	Mcat	0.425572101	0.02455785
ENSRNOP00000020675	ATP synthase membrane subunit c locus 2	Atp5mc2	0.401183768	0.04950002
ENSRNOP00000005428	ras homolog family member T1	Rnf135	0.398680297	0.006928878

ENSRNOP00000010763	PX domain containing serine/threonine kinase	Pxk	0.376392822	0.003686184
ENSRNOP00000028811	peroxisomal biogenesis factor 11 beta	Pex11b	0.355224183	0.000881305
ENSRNOP00000039176	torsin 1A interacting protein 2	Tor1aip2	0.352787435	0.008814947
ENSRNOP00000004047	dynein, axonemal, heavy chain 17	Dnah17	0.336323954	0.003401464
ENSRNOP00000020704	asporin	Aspn	0.336110576	0.045890156
ENSRNOP00000062585	metallothionein 2A	Mt2A	0.335277843	0.000391398
ENSRNOP00000046415	vitronectin	Vtn	0.292705567	0.010773549
ENSRNOP00000018872	jade family PHD finger 1	Jade1	0.107207573	0.00021068

Tabel S2 216 differencially expressed proteins on collagen-induced PEVs . (P-value < 0.05 and fold change > 2).

Protein	Protein Name	Gene Name	ratio（Collagen/Control）	p-value
ENSRNOP00000045630	TAP binding protein	Tapbp	33.99417652	0.001344528
ENSRNOP00000007223	SAC1 like phosphatidylinositide phos	Sacm1l	18.71131641	0.038172963
ENSRNOP00000014704	solute carrier family 25 member 4	Slc25a4	17.6218526	0.048024315
ENSRNOP00000001738	ATPase sarcoplasmic/endoplasmic retic	Atp2a2	17.5310842	0.04313125
ENSRNOP00000037343	Cas scaffold protein family member 4	Cass4	16.95878645	0.02137159
ENSRNOP00000061946	ATP synthase F1 subunit gamma	Atp5f1c	13.1333662	0.041357177
ENSRNOP00000027360	major vault protein	Mvp	13.04881625	0.036011989
ENSRNOP00000027445	myosin light chain 9	Myl9	12.43148756	0.0094766
ENSRNOP00000010218	prostaglandin-endoperoxide synthase 1	Ptgs1	12.40022232	0.009885799
ENSRNOP00000005846	dishevelled associated activator of mor	Daam1	11.85462818	0.047160418

ENSRNOP00000034921	citrate synthase	Cs	11.60964661	0.003016871
ENSRNOP00000065722	solute carrier family 14 member 1	Slc14a1	11.36536485	0.025171539
ENSRNOP00000014382	peptidylprolyl isomerase F	Ppif	10.74328917	0.015957974
ENSRNOP00000051866	integrin subunit alpha X	Itgax	10.69496844	0.038728267
ENSRNOP00000005719	microsomal glutathione S-transferase	Mgst3	10.53171966	0.001864867
ENSRNOP00000070305	B-cell receptor-associated protein 3	Bcap31	10.49202899	0.01448631
ENSRNOP00000066002	ribophorin I	Rpn1	9.586485649	0.040841695
ENSRNOP00000024033	cytochrome c oxidase subunit 4i1	Cox4i1	9.515397074	0.030683571
ENSRNOP00000010573	acetyl-CoA acetyltransferase 1	Acat1	9.373822358	0.018328586
ENSRNOP00000040859	calnexin	Canx	8.793270461	0.045891913
ENSRNOP00000010185	ADP-ribosylation factor like GTPase	Arl6ip5	8.533711794	0.019496498
ENSRNOP00000006443	reticulon 4	Rtn4	8.471474613	0.020542231
ENSRNOP00000068700	heat shock protein family E (Hsp10) mem	Hspe1	7.766508615	0.047897601
ENSRNOP00000015102	isocitrate dehydrogenase (NAD(+)) 3 cat	Idh3a	7.713411499	0.049838217
ENSRNOP00000017090	lysophosphatidylcholine acyltransfer	Lpcat3	7.620941196	0.015920675
ENSRNOP00000008477	voltage-dependent anion channel 1	Vdac1	7.535767005	0.012473471
ENSRNOP00000037703		AABR07069219.1	7.515306855	0.021442108
ENSRNOP00000048723	cytochrome c oxidase subunit 6B1	Cox6b1	7.489173573	0.019007078
ENSRNOP00000035440	myosin heavy chain 9-like 1	Myh9l1	7.405954119	0.034027704
ENSRNOP00000071802	thioredoxin-related transmembrane protei	Tmx1	7.061298809	0.009166453
ENSRNOP00000026783	caveolae associated protein 1	Cavin1	7.036983062	0.007092393
ENSRNOP00000021048	myosin light chain 12A	Myl12a	6.935494577	0.009394466

ENSRNOP0000 0005987	clathrin heavy chain	Cltc	6.829476466	0.03592 7537
ENSRNOP0000 0073838	myosin light chain 6	Myl6	6.792152577	0.03826 2578
ENSRNOP0000 0044296	actin, beta	Actb	6.660572749	0.00455 1156
ENSRNOP0000 0044055	protein tyrosine phosphatase, recepto	Ptpro	6.518122487	0.00680 2522
ENSRNOP0000 0070614	calpain 5	Capn5	6.468205327	0.02241 1136
ENSRNOP0000 0071314	mitochondrial calcium uniporter	Mcu	6.459501548	0.00546 3883
ENSRNOP0000 0008905	transient receptor potential cation chann	Trpc6	6.267364227	0.01937 4906
ENSRNOP0000 0022892	ATP synthase F1 subunit alpha	Atp5f1a	6.224370063	0.03605 6884
ENSRNOP0000 0059185	secretory carrier membrane protein 2	Scamp2	6.079855103	0.01358 5426
ENSRNOP0000 0063666	heat shock protein family D (Hsp60) me	Hspd1	6.058863837	0.00736 7303
ENSRNOP0000 0031564	tubulin, alpha-like 3	Tubal3	5.9649961	0.00584 1204
ENSRNOP0000 0025904	stomatin	Stom	5.922016761	0.02702 8789
ENSRNOP0000 0072636	myosin heavy chain 14	Myh14	5.781351281	0.03753 6076
ENSRNOP0000 0074839	voltage-dependent anion channel 2	Vdac2	5.743978181	0.00784 8944
ENSRNOP0000 0003965	ATP synthase F1 subunit beta	Atp5f1b	5.735062148	0.00527 4217
ENSRNOP0000 0039072	kalirin, RhoGEF kinase	Kalrn	5.317904367	0.01943 2257
ENSRNOP0000 0024068	purinergic receptor P2X 1	P2rx1	5.166502226	0.01934 0532
ENSRNOP0000 0015186	peroxiredoxin 3	Prdx3	4.973646049	0.01374 6396
ENSRNOP0000 0071860	phospholipase A1 member A	Pla1a	4.934535064	0.03476 6849
ENSRNOP0000 0013759	nipsnap homolog 3B	Nipsnap3b	4.900179896	0.03992 9086
ENSRNOP0000 0072442	acyl-CoA dehydrogenase family, membe	Acad11	4.899259967	0.04984 0142
ENSRNOP0000 0007567	NDUFA4, mitochondrial complex associa	Ndufa4	4.786435044	0.02461 1029

ENSRNOP0000<br>0061516	actin related protein 2/3<br>complex, sub	Arpc5	4.720270902	0.01877<br>7888
ENSRNOP0000<br>0067418	EMAP like 1	Eml1	4.539314834	0.00857<br>5905
ENSRNOP0000<br>0001958	malate dehydrogenase 2	Mdh2	4.508097472	0.03463<br>6058
ENSRNOP0000<br>0025828	RAB18, member RAS<br>oncogene family	Rab18	4.465535566	0.00322<br>235
ENSRNOP0000<br>0068338	selectin P	Selp	4.388885806	0.04525<br>3395
ENSRNOP0000<br>0010932	elastin microfibril interfacer 1	Emilin1	4.333642011	0.04795<br>1628
ENSRNOP0000<br>0066684	solute carrier family 2<br>member 3	Slc2a3	4.279602559	0.02192<br>4633
ENSRNOP0000<br>0025902	protein kinase C, theta	Prkcq	4.24881265	0.02236<br>3552
ENSRNOP0000<br>0072273	actin, gamma 1<br>propeptide-like	LOC100361<br>457	4.246267603	0.00022<br>216
ENSRNOP0000<br>0006961	integrin subunit alpha V	Itgav	4.234428219	0.03430<br>7062
ENSRNOP0000<br>0004520	actin related protein 3	Actr3	4.233937569	0.04657<br>506
ENSRNOP0000<br>0068946	RAB27A, member RAS<br>oncogene family	Rab27a	4.197086377	0.01311<br>4738
ENSRNOP0000<br>0002550	synaptosome associated<br>protein 29	Snap29	4.076250128	0.02183<br>6017
ENSRNOP0000<br>0073053	mitochondrial antiviral<br>signaling prot	Mavs	4.017043037	0.04839<br>3989
ENSRNOP0000<br>0006284	trans-L-3-hydroxyproline<br>dehydratase	L3hypdh	4.010087685	0.01256<br>2372
ENSRNOP0000<br>0074919	actin related protein 2/3<br>complex, subu	Arpc2	3.92386465	0.04213<br>3322
ENSRNOP0000<br>0069574	creatine kinase,<br>mitochondrial 2	Ckmt2	3.901478011	0.00183<br>1164
ENSRNOP0000<br>0001490	tescalcin	Tesc	3.898689231	0.04685<br>7946
ENSRNOP0000<br>0015692	aquaporin 1 (Colton blood<br>group)	Aqp1	3.841954496	0.00086<br>4988
ENSRNOP0000<br>0071905	anoctamin 6	Ano6	3.835984406	0.02843<br>2914
ENSRNOP0000<br>0004909	synaptotagmin-like 4	Sytl4	3.829271423	0.04645<br>3736
ENSRNOP0000<br>0016369	RAB27B, member RAS<br>oncogene family	Rab27b	3.81177141	0.00364<br>7773

ENSRNOP0000 0021318	syntaxin 7	Stx7	3.794388888	0.04763 724
ENSRNOP0000 0072041	capping actin protein of muscle Z-line	Capza2	3.774615676	0.03782 0599
ENSRNOP0000 0018138	triggering receptor expressed on myel	Treml1	3.748344575	0.01549 1121
ENSRNOP0000 0026197	voltage-dependent anion channel 3	Vdac3	3.688562154	0.01847 8627
ENSRNOP0000 0070464	protein tyrosine phosphatase, receptor	Ptprj	3.67574876	0.02123 9179
ENSRNOP0000 0022827	phospholipase A2 group IIA	Pla2g2a	3.592736676	0.04625 3074
ENSRNOP0000 0050701	RT1 class Ia, locus A2	RT1-A2	3.584917551	0.01248 0849
ENSRNOP0000 0025649	RAB14, member RAS oncogene family	Rab14	3.556076345	0.01707 4916
ENSRNOP0000 0035547	lymphocyte antigen 6 family member G6F	Ly6g6f	3.532685312	0.02630 1314
ENSRNOP0000 0011937	actin related protein 2/3 complex, su	Arpc4	3.47621568	0.03248 3848
ENSRNOP0000 0054740	similar to 60S acidic ribosomal pr	LOC498555	3.457487671	0.00126 882
ENSRNOP0000 0058759	calpastatin	Cast	3.452164059	0.00022 8546
ENSRNOP0000 0030025	caveolae associated protein 2	Cavin2	3.434679744	0.03178 0191
ENSRNOP0000 0067271	integrin subunit beta 3	Itgb3	3.415816802	0.03265 9641
ENSRNOP0000 0065234	RAB10, member RAS oncogene family	Rab10	3.404919559	0.03861 6941
ENSRNOP0000 0070703	filamin A	Flna	3.400115098	0.03717 6927
ENSRNOP0000 0006141	F11 receptor	F11r	3.370825007	0.04687 4951
ENSRNOP0000 0022550	G protein subunit alpha i2	Gnai2	3.363741267	0.01184 1089
ENSRNOP0000 0011065	vesicle-associated membrane protein 7	Vamp7	3.350847514	0.02368 0972
ENSRNOP0000 0004091	calreticulin	Calr	3.327062629	0.04793 2682
ENSRNOP0000 0018190	RAS like proto-oncogene A	Rala	3.288006649	0.02248 1227
ENSRNOP0000 0074369	DnaJ heat shock protein family (Hsp40)	Dnajb2	3.284196629	0.00032 9478

ENSRNOP0000 0064401		AABR07053 516.1	3.212327357	0.04441 3399
ENSRNOP0000 0017232	fibrinogen-like 2	Fgl2	3.209489909	0.04611 5769
ENSRNOP0000 0048390	RHO family interacting cell polariza	Ripor2	3.200236756	0.00763 7078
ENSRNOP0000 0043399	adducin 3	Add3	3.195819547	0.01394 8463
ENSRNOP0000 0013446	serine incorporator 3	Serinc3	3.160822814	0.00362 2078
ENSRNOP0000 0040934	protein phosphatase 1, regulatory s	Ppp1r12a	3.116027001	0.00575 9606
ENSRNOP0000 0027809	RAS related	Rras	3.102037642	0.02149 0017
ENSRNOP0000 0034828	eukaryotic translation elongation fa	Eef1d	3.088878926	0.01538 1899
ENSRNOP0000 0002638	SCY1 like pseudokinase 2	Scyl2	3.069324034	0.03620 7148
ENSRNOP0000 0003087	SLAIN motif family, member 2	Slain2	3.056957405	0.03267 7384
ENSRNOP0000 0062762	sphingomyelin phosphodiesterase, a	Smpdl3b	3.012722456	0.02234 017
ENSRNOP0000 0073255	syntaxin 11	Stx11	2.962110354	0.03481 179
ENSRNOP0000 0018899	PDZ and LIM domain 7	Pdlim7	2.95052026	0.03516 2371
ENSRNOP0000 0010197	RAB11B, member RAS oncogene family	Rab11b	2.913753856	0.03048 5672
ENSRNOP0000 0027407	cathepsin D	Ctsd	2.869029751	0.00631 9501
ENSRNOP0000 0067788	RAB1B, member RAS oncogene family	Rab1b	2.862783649	0.04200 1945
ENSRNOP0000 0073673	CD151 antigen-like	LOC100911 730	2.788854946	0.02932 5284
ENSRNOP0000 0037803	ferritin heavy chain 1	Fth1	2.780557418	0.04096 0686
ENSRNOP0000 0072730	golgi reassembly stacking protein 2	Gorasp2	2.777002129	0.04957 9479
ENSRNOP0000 0018864	EF-hand domain family, member D2	Efhd2	2.720003434	0.04316 0529
ENSRNOP0000 0024261	septin 2	Septin2	2.700162638	0.03835 7446
ENSRNOP0000 0068563		AABR07030 861.1	2.677638713	0.04003 2895

ENSRNOP0000 0060222	cyclin-dependent kinase 16	Cdk16	2.62649661	0.02633 5435
ENSRNOP0000 0035433	multimerin 1	Mmrn1	2.526396112	0.02845 8946
ENSRNOP0000 0073493	RAB1A, member RAS oncogene family	Rab1a	2.481461082	0.01442 6196
ENSRNOP0000 0072800	coagulation factor V	F5	2.455006273	0.04268 3698
ENSRNOP0000 0000673	inorganic pyrophosphatase 1	Ppa1	2.454292305	0.00599 1225
ENSRNOP0000 0012847	chaperonin containing TCP1 subunit 4	Cct4	2.450196142	0.04369 623
ENSRNOP0000 0022573	DnaJ heat shock protein family (Hsp40)	Dnaja2	2.444314934	0.03002 9617
ENSRNOP0000 0003413	RAS like proto-oncogene B	Ralb	2.398433735	0.00248 5066
ENSRNOP0000 0057378		AABR07033 987.1	2.394513849	0.04489 0499
ENSRNOP0000 0016432	RAB7A, member RAS oncogene family	Rab7a	2.393688522	0.01579 1116
ENSRNOP0000 0001779	G protein subunit alpha z	Gnaz	2.372826961	0.01224 2606
ENSRNOP0000 0001638	PTTG1 interacting protein	Pttg1ip	2.348884263	0.01041 6298
ENSRNOP0000 0030596	clathrin interactor 1	Clint1	2.31320912	0.00091 6914
ENSRNOP0000 0052051	integrin subunit alpha 2b	Itga2b	2.312092073	0.02372 0071
ENSRNOP0000 0068435	megakaryocyte and platelet inhibitory	Mpig6b	2.292245247	0.03502 5542
ENSRNOP0000 0008163	ADP-ribosylation factor like GTPase 8A	Arl8a	2.156035656	0.03085 2043
ENSRNOP0000 0051938	G protein subunit alpha 13	Gna13	2.130329251	0.02666 0802
ENSRNOP0000 0001911	G protein subunit beta 2	Gnb2	2.128994182	0.01121 4284
ENSRNOP0000 0067224		AABR07011 951.1	2.097979619	0.01608 0841
ENSRNOP0000 0068843	protein phosphatase 1, regulatory	Ppp1r3d	2.069581752	0.03874 3946
ENSRNOP0000 0013026	NUMB, endocytic adaptor protein	Numb	2.017697954	0.04608 7272
ENSRNOP0000 0025064	heat shock protein family A (Hsp70) mem	Hspa5	2.005645514	0.01294 2842

ENSRNOP00000060007	fibrinogen alpha chain	Fga	0.497950204	0.024085768
ENSRNOP00000032735	fibrinogen gamma chain	Fgg	0.48184529	0.04129625
ENSRNOP00000046415	vitronectin	Vtn	0.477982839	0.044441863
ENSRNOP00000069751	kelch-like family member 23	Klhl23	0.476643936	0.041253028
ENSRNOP00000073777	myosin IG	Myo1g	0.475826029	0.012226685
ENSRNOP00000046261		AABR07065789.3	0.47086594	0.048390845
ENSRNOP00000003748	serpin family C member 1	Serpinc1	0.453075908	0.038681228
ENSRNOP00000037902	complement C4B (Chido blood group)	C4b	0.444131104	0.025373369
ENSRNOP00000073812	kininogen 1	Kng1	0.425130465	0.008234705
ENSRNOP00000020196	haptoglobin	Hp	0.422469532	0.043963163
ENSRNOP00000014073	Serine protease inhibitor	LOC299282	0.417676218	0.041935923
ENSRNOP00000003921	albumin	Alb	0.415021129	0.049605388
ENSRNOP00000013896	serine (or cysteine) proteinase	Serpina3c	0.411474687	0.020560548
ENSRNOP00000058007	inter-alpha-trypsin inhibitor heavy chai	Itih4	0.405261203	0.004167549
ENSRNOP00000009813	fibrinogen beta chain	Fgb	0.403287709	0.008016009
ENSRNOP00000022233	coagulation factor II	F2	0.394043255	0.039056318
ENSRNOP00000007061	thyroid hormone receptor interactor	Trip11	0.393453924	0.046989674
ENSRNOP00000050806		AABR07065823.2	0.378945083	0.037231382
ENSRNOP00000064737	protein S	Pros1	0.352492903	0.023063113
ENSRNOP00000013175	serine (or cysteine) proteinase	Serpina3m	0.351380051	0.004540122
ENSRNOP00000009248	alpha-1-microglobulin/bikunin precursor	Ambp	0.351258049	0.003872828
ENSRNOP00000026677	coagulation factor X	F10	0.350742322	0.017266705

ENSRNOP0000 0017195	complement factor H-related 1	Cfhr1	0.346202818	0.02677 9601
ENSRNOP0000 0065880	similar to hepatic multiple inosi	RGD156480 1	0.34384458	0.02900 3742
ENSRNOP0000 0028740	extracellular matrix protein 1	Ecm1	0.34088104	0.04524 1903
ENSRNOP0000 0004322	serpin family F member 2	Serpinf2	0.334876261	0.01698 7759
ENSRNOP0000 0005155	superoxide dismutase 3	Sod3	0.334004956	0.01758 1237
ENSRNOP0000 0051504	fetuin B	Fetub	0.329775052	0.02847 4202
ENSRNOP0000 0055223		AABR07034 632.1	0.322177653	0.00088 2549
ENSRNOP0000 0051714	EH-domain containing 1	Ehd1	0.319120292	0.02043 833
ENSRNOP0000 0029319		AABR07060 872.1	0.315380272	0.03755 4833
ENSRNOP0000 0074387	complement C7	C7	0.31367774	0.00871 2593
ENSRNOP0000 0063871	complement C8 alpha chain	C8a	0.309454051	0.01028 0915
ENSRNOP0000 0019237	kallikrein B1	Klkb1	0.308723494	0.04237 9436
ENSRNOP0000 0024917	angiotensinogen	Agt	0.306120524	0.03892 9732
ENSRNOP0000 0064104	protein Z-dependent protease	LOC100909 524	0.305087209	0.01989 0689
ENSRNOP0000 0065206	apolipoprotein A1	Apoa1	0.3007088	0.04034 9849
ENSRNOP0000 0023984	inter-alpha trypsin inhibitor, heavy ch	Itih3	0.294255295	0.00969 1826
ENSRNOP0000 0024196	glycosylphosphatidylinositol specific	Gpld1	0.292861592	0.00169 7733
ENSRNOP0000 0015723	mannose-binding lectin (protein A) 1	Mbl1	0.289185269	0.04889 2554
ENSRNOP0000 0015318	protein phosphatase 2, regulatory s	Ppp2r2a	0.288841299	0.00835 2212
ENSRNOP0000 0041165	inter-alpha trypsin inhibitor, heavy ch	Itih1	0.288433109	0.02613 8883
ENSRNOP0000 0009817	serpin family G member 1	Serping1	0.282072678	0.03204 699
ENSRNOP0000 0018545	complement C9	C9	0.280589541	0.01778 7146

ENSRNOP00000066273		AABR07065823.3	0.280394233	0.007943301
ENSRNOP00000074115	coagulation factor XII	F12	0.279854027	0.046169553
ENSRNOP00000001826	HECT domain E3 ubiquitin protein lig	Hectd4	0.276635697	0.045823997
ENSRNOP00000071202		AABR07060886.1	0.275471252	0.0366212
ENSRNOP00000004559	coagulation factor IX	F9	0.274504119	0.015827142
ENSRNOP00000026666	protein Z, vitamin K-dependent plasma	Proz	0.268168684	0.017705627
ENSRNOP00000006109	lumican	Lum	0.265993438	0.024845841
ENSRNOP00000026583	lecithin cholesterol acyltransferase	Lcat	0.261240431	0.003063581
ENSRNOP00000072149		AABR07065699.4	0.261006699	0.046685912
ENSRNOP00000017385	complement C1q A chain	C1qa	0.260784373	0.03025344
ENSRNOP00000065605		AABR07051652.1	0.255203096	0.031106865
ENSRNOP00000073074		AABR07051689.1	0.255155818	0.026783871
ENSRNOP00000028656	epithelial membrane protein 3	Emp3	0.238197635	0.02791634
ENSRNOP00000075643	coagulation factor XIII B chain	F13b	0.230853481	0.032415114
ENSRNOP00000045637	transferrin	Tf	0.230731319	0.030541832
ENSRNOP00000016330	complement C1s	C1s	0.228479308	0.015208707
ENSRNOP00000017065	complement C1q C chain	C1qc	0.228467967	0.047184978
ENSRNOP00000031829	serpin family D member 1	Serpind1	0.2278647	0.011662873
ENSRNOP00000011823	paraoxonase 1	Pon1	0.226184643	0.014827044
ENSRNOP00000001801	alpha-2-glycoprotein 1, zinc-binding [	Azgp1	0.225715031	0.038506787
ENSRNOP00000010100	complement C8 beta chain	C8b	0.223604597	0.012121776
ENSRNOP00000070684	Serine protease inhibitor	LOC299282	0.223120759	0.021224278

ENSRNOP00000002645	interleukin 1 receptor accessory pro	Il1rap	0.214260068	0.033079072
ENSRNOP00000012500	serpin family A member 6	Serpina6	0.201655259	0.021675319
ENSRNOP00000064165	peptidoglycan recognition protein 2	Pglyrp2	0.178875143	0.023168483
ENSRNOP00000028847	attractin	Atrn	0.166601173	0.033854607
ENSRNOP00000016317	mannan-binding lectin serine peptidas	Masp2	0.160162406	0.005112157
ENSRNOP00000001126	apolipoprotein M	Apom	0.146641874	0.041047478
ENSRNOP00000013845	DENN domain containing 10	Dennd10	0.139023974	0.013818273
ENSRNOP00000002507	mannan-binding lectin serine peptidase	Masp1	0.118583576	0.021708471
